# Respiratory syncytial virus fuses with plasma membrane to infect primary cultures of bronchial epithelial cells

**DOI:** 10.3389/fmicb.2025.1498955

**Published:** 2025-02-26

**Authors:** Christian Cadena-Cruz, Marcio De-Avila-Arias, Heather M. Costello, Leidy Hurtado-Gomez, Walter Martínez-De-La-Rosa, Gigliola Macchia-Ceballos, Wendy Rosales-Rada, Gerardo Valencia-Villa, Pedro Villalba-Amarís, Meisam Naeimi Kararoudi, Mark E. Peeples, Homero San-Juan-Vergara

**Affiliations:** ^1^Departamento de Medicina, Universidad del Norte, Barranquilla, Colombia; ^2^Programa de Bacteriología, Universidad Libre Seccional, Barranquilla, Colombia; ^3^Center for Vaccines and Immunity, Abigail Wexner Research Institute at Nationwide Children’s Hospital, Columbus, OH, United States; ^4^Grupo de Investigación Avanzada en Biomedicina, Programa de Microbiología, Universidad Libre de Colombia, Barranquilla, Atlántico, Colombia; ^5^Center for Childhood Cancer and Blood Diseases, Abigail Wexner Research Institute at Nationwide Children’s Hospital, Columbus, OH, United States; ^6^Department of Pediatrics, The Ohio State University College of Medicine, Columbus, OH, United States; ^7^Infectious Disease Institute, The Ohio State University, Columbus, OH, United States

**Keywords:** respiratory sycytial virus, synchronization, plasma membrane, viral entry, endocytosis

## Abstract

**Background:**

Respiratory syncytial virus (RSV) is a common cause of bronchiolitis in children under the age of five. RSV infection proceeds by fusion of the viral envelope with the target cell membrane, but it is unclear whether fusion occurs with plasma or endosomal membranes.

**Methods:**

Entry and/or infection was studied in undifferentiated primary cultures of human bronchial epithelial cells. Synchronization of viral entry or infection was achieved by attaching the virus to the plasma membrane at temperatures of 4°C or 22°C. Cells in which entry events had occurred were identified by the enzymatic action of beta-lactamase M (BlaM) fused to the RSV P protein (BlaM-P) carried by rgRSV virions. BlaM cleaves the beta-lactam ring of CCF2 loaded into the cells, disrupting FRET and allowing blue light to be emitted. Green fluorescent protein (GFP) expression, encoded by the rgRSV genome, was used to identify infected cells.

**Results:**

We found that adsorption of RSV at 4°C favors entry via endocytosis, whereas binding of the virus to the membrane at 22°C favors RSV entry via the plasma membrane. The induction of endocytosis by synchronization at 4°C is, therefore, an artifact. In addition, we found that all drugs that interfered with RSV infection reduced cell membrane deformations such as filopodia and lamellipodia, suggesting a mechanism by which they may interfere with RSV fusion with the cell membrane.

**Discussion:**

In conclusion, RSV enters the cell by direct fusion of its envelope with the plasma membrane.

## Introduction

Respiratory syncytial virus (RSV) is a non-segmented, negative sense RNA virus that is the type species of the family Pneumoviridae (Collins et al., 2013). RSV is a leading cause of acute lower respiratory tract infection in infants (bronchiolitis and pneumonia) and the elderly (pneumonia) (Mazur et al., 2018). Furthermore, RSV infection is a major cause of acute exacerbations of chronic obstructive pulmonary disease in older adults and is a significant contributor to morbidity and mortality in this group (Hosseini et al., 2015; Kokturk et al., 2015).

The RSV envelope is derived from the infected cell membrane and contains the three glycoproteins encoded by the virus (Collins and Melero, 2011; McLellan et al., 2013b). The SH protein may be a viroporin (Triantafilou et al., 2013) and confers resistance to the action of tumor necrosis factor-alpha (TNF-alpha) (Fuentes et al., 2007). The G glycoprotein mediates attachment (McLellan et al., 2013b). The trimeric F glycoprotein triggers fusion between the virion and the target cell membranes (McLellan et al., 2013a). Activation of the RSV F protein requires cleavage at 2 furin consensus sequences; FCS1 (RARR109) and FCS2 (KKRKRR136). The fusion peptide is immediately downstream to FCS2 (Gilman et al., 2015). After proteolytic processing, a minor peptide (p27) is released. There is strong evidence that cleavage at both sites occurs within the same time frame, soon after protein synthesis, so that virions may bud with a fraction of trimeric, fully cleaved pre-F fusion protein (Neal et al., 2024; Zimmer et al., 2001; Rezende et al., 2023). The efficiency of p27 cleavage depends on the duration of infection, the RSV strain and the cells used for virus production (Korabel et al., 2021).

Several proteins in the target cell membrane interact with either the G-or F-proteins. In immortalized cells, annexin-2 (Malhotra et al., 2003) and heparan sulfate-rich proteoglycans (Hallak et al., 2007) have been identified as G protein receptors. In well-differentiated primary human airway epithelial cultures, the *ex vivo* model of airway infection, CX3CR1 and/or CCR3 appear to be the receptor(s) for the G protein (Johnson et al., 2015; Jeong et al., 2015; Chirkova et al., 2015; Tripp et al., 2001; Wellemans et al., 2021). Potential receptors for the F protein include nucleolin or ICAM-I (Behera et al., 2001; Tayyari et al., 2011; Mastrangelo and Hegele, 2013).

Once an RSV virion contacts the cell membrane, it likely glides or tumbles over the cell plasma membrane as a result of G protein binding to potential receptors embedded in cholesterol-rich membrane domains (Alonas et al., 2014; San-Juan-Vergara et al., 2012), but the exact site where fusion occurs remains unclear.

Srinivasakumar et al. (1991) suggested that RSV fuses its envelope with the plasma membrane by measuring the diffusion of R18 during hemifusion. Razinkov et al. (2001) adsorbed R18-labeled RSV at both 4°C and 37°C to investigate which viral entry event was affected by the fusion inhibitor RFI-641, however, the experimental design did not assess where lipid mixing occurred; therefore, they cannot determine the location where RSV fuses its membrane with the membrane of cell.

Kolokoltsov et al. (2007) using siRNA to reduce the expression of clathrin pathway-associated molecules dramatically reduced RSV infection, suggesting that entry occurs via this endocytic pathway.

Five previous reports have explored RSV entry at 37°C after binding the virus to the cell membrane at 4°C. Zheng et al. (2014) prepared dual-labeled RSV virions, in which the envelope and the genome were labeled with biotin-bound streptavidin and an RNA-binding fluorochrome, respectively. Upon warming to 37°C, they suggested that the signal decoupling occurred in the cytosol, close to the plasma membrane. However, they were unable to determine where the viral content was released due to the limitations of confocal microscopy. Spots separated by less than 250 nm cannot be distinguished from each other. In a subsequent study, Zheng et al. (2017) showed the presence of virions within macropinosomes after adsorption at 4°C and subsequent temperature shift to 37°C.

Following a strategy developed by Sakai et al. (2006), the differential diffusion rates of R18 and DiOC18 from dual-labeled virions led San-Juan-Vergara et al. (2012), Krzyzaniak et al. (2013), and Chen et al. (2020) to conclude that RSV fused its envelope to the endosomal membrane. San-Juan-Vergara et al. (2012) and Krzyzaniak et al. (2013) found that virions were internalized via macropinosomes and that dequenching of fluorescently labeled virions occurred in endosomes. Krzyzaniak et al. (2013) showed that a proteolytic process in the endosome is required to cleave the FCS2, since their viral preparations were enriched in RSV F proteins that were cleaved only at FCS1, leaving p27 still attached to the fusion peptide. However, this may be an extreme case of inefficient cleavage (Rezende et al., 2023).

Lingemann et al. (2019) reported that RSV infection seemed to depend on ATPase Na^+^/K^+^ Transporting Subunit Alpha 1 (ATP1A1), which they proposed facilitates virion uptake via macropinocytosis. However, they did not conduct functional assays, and thus, they could not determine whether the reduction in RSV infection was due to impairing RSV fusion at the plasma membrane or its endocytic uptake. Kieser et al. (2023) reported that during RSV entry, cells underwent cytoskeletal changes consistent with macropinocytosis and that virions appeared to colocalize with early endosomes 90 min after rewarming cell cultures to 37°C following 1 h of viral synchronization on ice.

These reports also showed that drugs impairing macropinocytosis significantly reduced RSV infection and/or entry. Overall, these reports show that virions adsorbed at 4°C for synchronization enter by macropinocytosis upon warming to 37°C.

Here we have engineered RSV to express the beta-lactamase (BlaM) reporter protein fused to the viral phosphoprotein (P). BlaM activity allows rapid detection of the cytoplasmic delivery of viral contents, and therefore, viral entry. Using a combination of kinetic assays to distinguish between the entry of viruses still attached to the cell surface and those entering from endosomes at each time point, we found that our data imply that RSV virions may also deliver their contents by fusing their envelope to the plasma membrane at 37°C. When virus was attached at a temperature as low as 4°C, the traditional approach for assessing virus entry, warming to 37°C induced changes in the plasma membrane that facilitated endocytosis of cell membrane-adsorbed virions. In addition, we found that all drugs that interfere with RSV infection reduced cell membrane deformations such as filopodia and lamellipodia, suggesting a mechanism by which they may interfere with RSV fusion with the cell membrane.

## Materials and methods

### Cell culture

We acquired undifferentiated normal human bronchial epithelial (NHBE) cells from Lonza, while murine fibroblasts—3T3-J2—were purchased from Kerafast. We used conditionally reprograming for a continuous expansion of NHBE cells in order to take the number of passages beyond the critical point of senescence (Palechor-Ceron et al., 2013). For such conditional reprogramming, undifferentiated NHBE cells were grown on a bed of mitomycin-treated 3T3-J2 fibroblast cells in the presence of the ROCK inhibitor, Y27632 (Tocris).

For 3T3-J2 cell expansion, we cultured them in 3T3 medium: DMEM (Thermo Fisher Scientific) supplemented with 10% bovine calf serum (Thermo Fisher Scientific) and 1% penicillin-streptomycin (Thermo Fisher Scientific). Cells were incubated at 37°C with 5% CO_2_, and 95% relative humidity until confluency reached 70%. Cells were then seeded in new T-175 flasks (Corning) at a density of 3.5 × 10^3^ cells per cm^2^. After 80 h, cell monolayers were washed with 1X PBS followed by adding fresh 3T3-J2 media supplemented with 2 μg/mL mitomycin C (Sigma Aldrich) to inactivate mitosis. After removing mytomicin-supplemented 3T3 medium, we carefully washed then the cell monolayer with 1X PBS thrice to remove mitomycin excess. Then, cell monolayers were detached from the surface using trypsin (Sigma Aldrich). After trypsin neutralization and cell washing and pelleting by centrifugation, cells were seeded at a density of 2 × 10^4^ cells/cm^2^ in T75 or T175 flasks coated with collagen. After overnight incubation for cell adhesion, the mytomicin-treated 3T3-J2 cells were ready to support the expansion of epithelial cells.

Primary undifferentiated NHBE cells were seeded on mytomicin-treated 3T3-J2 bed in F medium supplemented with the ROCK inhibitor Y-27632 (7.5 μM), as previously described by Liu et al. (2012). Cultures were kept at 37°C with 5% CO_2_ and 95% relative humidity. NHBE cells began to displace 3T3-J2 cells and at the same time formed round colonies. Once they reached 70% confluence, we incubated the culture in PBS + EDTA (Dulbecco’s 1X PBS; 100 mM EDTA) for 5 min to detach the 3T3-J2 fibroblasts. Then, NHBE cells were detached using a Trypsin-based commercial kit from Lonza following respective recommendations. Finally, NHBE cells were cryo-preserved in F medium supplemented with 10% FBS, 10% DMSO and 7.5 μM ROCK inhibitor.

For experimental assays, the undifferentiated NHBE cells were cultured and expanded in BEGM medium (Lonza) supplemented with 7.5 μM ROCK inhibitor in T75 flasks for 3 days. Then, NHBE cells were sub-cultured and seeded in 24-well plates at a density of 1.2 × 10^5^ cells/well in the presence of ROCK inhibitor. After overnight incubation, we removed Y27632 by changing the medium. After 24 h incubation in BEGM, cells reached 80% confluency and we used them for viral infection assays.

### Assaying the F-actin state by staining with Alexa-488-labeled phalloidin

We evaluated whether temperature may have an impact on the actin cytoskeleton by staining F-actin with phalloidin. Briefly, NHBE cells were subcultured on rounded polylysine-coated glass coverslips (PDL, Neuvitro). At 70% confluency, cell cultures were incubated at different temperatures (4°C, 22°C or 37°C) for 1 h. A different set of cultures were used to explore the effect of a sudden temperature change on the F-actin cytoskeleton. This was conducted by exposing cultures to either 4°C or 22°C, which was then followed by incubation at 37°C for 10 min. After temperature treatment, we fixed NHBE cell cultures with 4% paraformaldehyde for 15 min at 4°C, which was followed by permeabilization with Triton X-100 for 15 min at room temperature. Finally, we incubated each cell culture with Alexa-488-labeled phalloidin (Thermo Fisher Scientific) in order to tag F-actin. We used FluorSafe mounting medium (Calbiochem). The cells were visualized at 63× (NA 1.4) using a confocal microscopy Axio observer Z1. Using Zen-Blue software, multiple *Z*-plane frames were serially acquired with a separation of 0.5 μm from each other along the *Z*-axis using the module *Z*-stack. Then, the images were merged and subjected to signal deconvolution using the module extended depth of focus.

### Design, construction and production of rgRSV-P-BlaM

We used an RSV engineered to express BlaM as was reported by De Ávila-Arias et al. (2024). Briefly, we had the sequence encoding the chimera P-BlaM synthesized by Integrated DNA Technologies as gBlocks. P stands for the RSV phosphoprotein, and BlaM corresponded to the optimized beta lactamase version (Y105W) (Wolf et al., 2009). By Gibson cloning (New England Biolabs), we inserted the gBlock at the sequence flanked by AvrII (nt. 2930) and PvuI (nt. 6573), using HC123 full-length RSV cDNA construct as a backbone. HC123 was derived from the green fluorescent protein (GFP) expressing cDNA MP224 (Hallak et al., 2000). The modified HC123 cDNA construct sequence has P-BlaM positioned after the RSV-P gene.

We used BHK cells constitutively expressing T7-RNA polymerase (BHK/T7 cells) to rescue the rgRSV-P-BlaM virus as described. BHK/T7 cells were subcultured in 6-well plates in DMEM-Glutamax medium (Thermo Fisher Scientific) supplemented with 2% FBS (Thermo Fisher Scientific) and 1% Penicillin-Streptomycin (Thermo Fisher Scientific). At 70% confluency, BHK/T7 cells were transfected with P-BlaM RSV cDNA and helper plasmids (Hotard et al., 2012) using Lipofectamine 3000 (Thermo Fisher Scientific)—in the following quantities: 800 ng of modified HC123 cDNA vector, 400 ng of N, P, and M2-1, and 200 ng of L. Transcription of each plasmid was driven by the T7 promoter. Two days later, transfected cells were subcultured in T25 flask (Corning), and syncytia formation was monitored for 48 h, followed by subculturing in a T75 flask (Corning). We proceeded to change medium each 48 h until we observed 50–60% syncytia in the monolayer. Cells were then scraped in a small fraction of the supernatant. Both scraped cells and supernatant were placed in the same Falcon tube, followed by a subsequent centrifugation at 2,141 g for 10 min at 4°C to pellet the cell debris. Magnesium sulfate at 0.1 M was added to stabilize the virus suspension. We snap-froze 1-mL aliquots in dry ice. Each aliquot was stored at −150°C (Panasonic).

### Production and titration of recombinant virions (rgRSV-P-BlaM and rgRSV)

We grew recombinant respiratory syncytial virus (RSV) in HEp-2 cell cultures. Briefly, HEp-2 cells were expanded in Opti-MEM^™^ medium supplemented with Glutamax (Thermo Fisher Scientific), 10% FBS (Thermo Fisher Scientific) and 1% Penicillin-Streptomycin (Thermo Fisher Scientific) in T75 flasks for 3 days at standard culture conditions of 37°C, 5% CO_2_, and 95% relative humidity. Subsequently, cells were transferred to T175 flasks at such cell density to reach 60% confluency the next day. After washing with Ca^++^- and Mg^++^-free PBS, HEp-2 cells were infected with rgRSV at a multiplicity of infection (MOI) of 1 infectious viral particle per 10 cells (MOI of 0.1). The infection proceeded in Opti-MEM (Thermo Fisher Scientific) supplemented with 2% FBS for 48 h while monitoring cytopathic effects and the formation of syncytia. At that time, we changed medium and left the culture proceed for 20 h more. Then, we transferred approximately ¾ of the medium to a 50 mL Falcon tube. We scraped the cells in the medium that was left in each flask and transferred them to the same 50-mL Falcon tube. The cell debris was pelleted by centrifugation at 2,141 g at 4°C for 10 min in a Sorvall Legend Match 1.6 centrifuge. The infectious supernatant was transferred to a new pre-chilled, labeled Falcon tube. We supplemented the supernatant to stabilize the virions with the following compounds at the indicated final concentrations: 0.1 M MgSO_4_ (Sigma Aldrich), 0.1% human Albumin (Biotest) and 50 mM HEPES (Gibco). One-mL aliquots of supernatant in cryotubes were snap-frozen in dry ice. Subsequently, these were stored in a −150°C freezer (Panasonic).

We estimated the virus titer by preparing five-fold serial dilutions from stock in HEp-2 cell cultures, which were previously subcultured in 24-well plates. After inoculation for 2 h at 37°C, the inoculum was removed, replaced with fresh medium and incubated for 16 h at 37°C. Trypsin treatment was used to detach cells from the well surface. Using fluorescence-based flow cytometry, we quantified RSV-infected cells. Following Techaarpornkul et al. (2001) recommendations, we calculated the viral titer from that dilution that gave a MOI in the range of 0.05 to 0.1. The formula to calculate such MOI is: (% of infected cells × total number of cells × dilution factor)/(100 × volume of the infection aliquot).

### Assessment of palivizumab concentration to neutralize RSV

We tested a range of concentrations to determine the neutralization profile of palivizumab (AbbVie). The rgRSV (recombinant green fluorescent protein expressing RSV) virus was incubated with palivizumab for 1 h at 37°C before adding the mixture to NHBE cell monolayer. We tested the following antibody concentrations: 200, 12.5, 0.78, 0.195, and 0.048 μg/mL. These antibody solutions were prepared in BEGM medium. After inoculation, as described above, infection was allowed to proceed for 16 h at 37°C. Then, cell cultures were processed for fluorescence-based flow cytometry. Infected cells were identified by GFP expression.

### Assays for determining RSV entry kinetics after synchronizing viral entry by adsorption at different temperatures (4°C or 22°C)

After washing with HEPES-based saline solution (HBSS), NHBE cells at 80% confluency were infected with rgRSV-P-BlaM at different infectious doses (please see respective results section) suspended in BEBM. The virus adsorption proceeded at either 22°C or 4°C for 1 h to synchronize viral attachment.

After synchronization at the chosen temperature, unbound virions were removed and cells were rinsed with HBSS, and the plates were placed in the incubator at 37°C. At different time intervals (0, 10, 30, 45, 60, 90 and 120 min), we stopped the infection either by using palivizumab (antibody block) or by placing a selected plate at 4°C (temperature block, TB).

When we used BlaM activity on CCF2-AM to identify RSV-infected cells, we stopped the infection by either antibody (200 μg/mL palivizumab) neutralization or TB (4°C) treatment at the respective interval. When the last time interval was reached, we changed the medium on all plates to HBSS supplemented with 2 μM CCF2-AM and 200 μg/mL palivizumab in all culture plates. CCF2-AM (GeneBLAzer *in vivo* Detection Kit, Thermo Fisher Scientific) was loaded into cells by incubating all cultures for 3 h at 17°C. This approach is known as “time-of-addition BlaM” assay and is the standard procedure as was reviewed by Jones and Padilla-Parra (2016). Finally, cells were prepared for fluorescence-based flow cytometry. CCF2 is a compound made up of a hydroxycoumarin moiety linked by a beta-lactam ring bridge to fluorescein. In the absence of BlaM, the FRET mechanism is present: excitation at 405 nm causes emission at 520 nm. In the presence of BlaM, the FRET mechanism is lost as BlaM cleaves the beta-lactam ring releasing the hydroxycoumarin from the fluorescein: excitation at 405 nm causes emission at 447 nm.

We also ran a TB-chase assay in which after synchronizing rgRSV-P-BlaM adsorption at 4°C for 1 h, we added 200 μg/mL palivizumab to each culture when switching the temperature to 37°C. After each abovementioned time interval, we placed the respective plate at 4°C to prevent virus entry via the endosome. When the last time interval was reached (150 min), cells were processed as above for determining BlaM activity using CCF2-AM.

When we used GFP fluorescence as the readout, we stopped the infection by changing the rgRSV-containing medium to BEGM supplemented with the RSV-neutralizing antibody (200 μg/mL palivizumab) to neutralize active virions adsorbed to the plasma membrane. Then, we allowed the infection to proceed for 16 h before preparing the cells for fluorescence-based flow cytometry, using a BD FACS CANTO II.

### Localization of RSV virions in endosomal compartments using the tyramide signal amplification

NHBE cells were grown on Millicell EZ slides (Millipore). Cells were infected with rgRSV-GFP (MOI = 2), and incubated at 22°C for 2, 5 and 10 h. Unattached virions at the cell membrane were removed using a cold trypsin-EDTA solution (0.95 mg/mL). Then, cells were fixed with 4% paraformaldehyde and permeabilized with 0.05% saponin. Endogenous peroxidase was inactivated by treating the cells with 2% hydrogen peroxide for 45 min.

Cells were incubated for 1 h at room temperature with the following primary antibodies: mouse anti-RSV-N, rabbit anti-EEA1, rabbit anti-RAB5, rabbit anti-RAB7, and rabbit anti-RAB11. Cells were incubated for 1 h with Alexa-488-conjugated goat anti-rabbit antibodies to identify the primary rabbit antibodies targeting the endosomal markers. After washing with 1X PBS to remove the antibody excess, cells were incubated for 1 h with horseradish peroxidase (HRP)-conjugated goat anti-mouse antibody. Following a final wash with 1X PBS, cells were incubated with Alexa 555-labeled tyramide for 10 min, whose amplification buffer was supplemented with hydrogen peroxide. Nuclei were labeled with DAPI (NucBlue, Thermo Scientific). FluorSave (Millipore) mounting media was used for image visualization and long-term preservation.

Cell preparations were imaged using a motorized, inverted Axio-observer Z1 fluorescence microscopy (Zeiss). Images were analyzed using the COLOC-2 plugin of ImageJ software. The colocalization of viral particles with the endosomal markers was determined by the Manders’ coefficient being ≥0.5.

### Drug screening targeting early steps of virus infection

Primary cultures of undifferentiated bronchial epithelial cells grown at 80% confluency in 24-well plates were exposed to different concentrations of drugs that were chosen based on their capability to interfere with cell processes associated to endocytosis. Each drug was administered to the cell cultures before adding the viral inoculum (rgRSV at an MOI of 0.2) and kept in the culture media during infection for 2 h at 37°C. After washing with HBSS to remove unabsorbed virions, a solution of cold trypsin (0.0025 mg%) was added to the cell cultures for 8 min at 4°C to remove unfused virions. Trypsin solution was withdrawn and after HBSS washing, fresh media was added to cell cultures. Infection was allowed to proceed for 16 h at 37°C. GFP positive cells were quantified by flow cytometry.

In order to confirm that positive hits targeted just the early steps conducive to RSV infections, cell cultures grown to 80% confluency were inoculated with rgRSV for 2 h at 37°C before adding the respective positive hit, which was kept in the culture medium for 3 h at 37°C. After withdrawing the respective inhibitor and washing the cell culture with HBSS, fresh media was added back to the cell cultures and the infection was allowed to proceed for 16 h at 37°C. GFP positive cells were quantified by flow cytometry.

The cell cytotoxicity of each of the positive hits was evaluated in primary cultures of undifferentiated bronchial epithelial cells grown at 80% confluency. Each drug was kept in the culture medium for 3 h at 37°C. Then, the medium containing the respective inhibitor was withdrawn and the cell culture was washed with HBSS. Fresh BEGM medium was added to the cell culture for 30 min. Cells were detached from the plate using trypsin resulting in a single cell suspension prepared in binding buffer. Cell suspension was incubated with Alexa-647 labeled annexin V and SYTOX blue, which was immediately analyzed by flow cytometry. Cells were classified as viable (double negative), early apoptosis (positive annexin V), late apoptosis (positive annexin V and SYTOX blue) or dead cells (positive SYTOX blue).

### Time-of-drug addition assays

Primary cultures of undifferentiated bronchial epithelial cells grown at 80% confluency in 24-well plates were infected with either rgRSV-P-BlaM or rgRSV to evaluate the role that each drug played during RSV entry or infection, respectively. The infectious aliquot dose was an MOI of 2. Virions were incubated at 22°C for 1 h to synchronize attachment and infection was initiated by placing the plates at 37°C. At different times (0, 10, 30, 45, 60, 90, and 120 min), either the drug or palivizumab was added to the respective plate. The time at which the plate was placed at 37°C was labeled as T0 (time zero).

For virus entry, cells were loaded with CCF2-AM for 3 h at 17°C, and the drug was maintained in the loading buffer. For virus infection, all plates received palivizumab at the end of the assay before allowing the infection to proceed at 37°C for 16 h. Flow cytometry was used to quantify BlaM (+) or GFP (+) cells.

### Statistical analysis

All experiments were performed at least in triplicate and are presented as normalized values with the mean ± standard deviation (SD). For the different assays, an ANOVA was performed to determine differences between treatments. A subsequent one way *t*-test was used to identify specific treatments showing statistically significant differences. All analyses were conducted using GraphPad Prism (GraphPad Software 9, La Jolla, CA, United States). The level of significance was set at *p* < 0.05. ^*^p < 0.05, ^**^p < 0.01, and ^***^p < 0.001.

## Results

### Incubation of undifferentiated normal human bronchial epithelial cells at 4°C disrupts actin filaments and induces cell blebbing after warming to 37°C

Attaching virus at 4°C for 1 h is one of the most common methods to synchronize virus entry and infection. We used Alexa-488-labeled phalloidin as a probe for F-actin to determine whether incubation at different temperatures affects the cell cytoskeleton in NHBE cell cultures. For illustration, we showed each of the F-actin-associated structures in Supplementary Figure S1.

**Figure 1:**
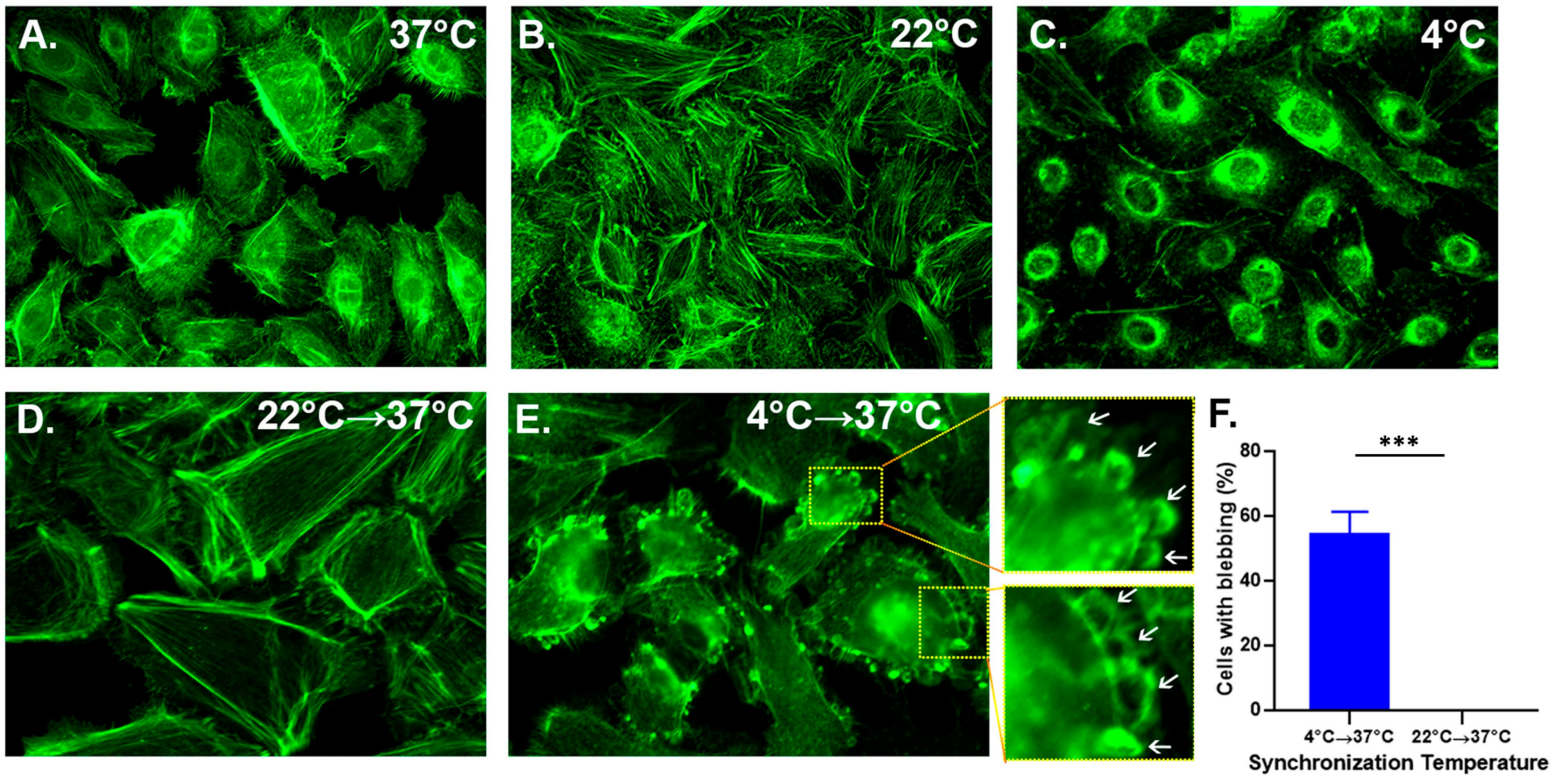
Cell membrane blebbing ensues spontaneously simply by increasing the temperature from 4°C to 37°C. NHBE cells were cultured on polylysine-coated coverslips until they reached 70% confluency. A set of coverslips were incubated at the indicated temperature for 1 h **(A–C)**, while in another set the last 10 min of the incubation was conducted at 37°C **(D,E)**. Cultures were fixed with 4% PFA, permeabilized and treated with Alexa 488-labeled phalloidin to identify the F-actin. The images were taken using a 63× objective (N.A. 1.4). A series of 10 images taken every 1 μm of focal plane in HeLa cells using the Z-stack in a Z-stack configuration in which each Z-section was 1 um. **(F)** Bar graph represents a quantitative evaluation of 10 fields of view (20–38 cells per field) showing cells developed actin rings when cultures were warmed. A one-way *t*-test was used for statistical analysis. The level of significance was set at *p* < 0.05; ^*^p < 0.05, ^**^p < 0.01, and ^***^p < 0.001.

At 37°C, the actin cytoskeleton of NHBE cells showed stress fibers, cortical actin, and perinuclear actin (Figure 1A). When NHBE cells were incubated at 22°C for 1 h, they showed stress fibers and cortical actin (Figure 1B). However, incubation at 4°C for the same time interval caused a collapse of F-actin structures, leaving only perinuclear actin (Figure 1C).

Ten minutes after shifting from 22°C to 37°C, no changes in the cytoskeleton were observed (Figure 1D vs. Figure 1B). In contrast, 10 min after shifting from 4°C to 37°C, actin rings under the plasma membrane were visible in most cells (Figure 1E). Quantitative evaluation of 10 different fields (20–38 cells per field) showed that 40–60% of all cells showed actin rings under the plasma membrane after warming from 4°C to 37°C. However, such rings were not visible after warming from 22°C to 37°C (Figure 1F). Actin rings are one of the features of membrane blebbing, the outward ballooning of the plasma membrane (Charras et al., 2006; Chikina et al., 2019). Consequently, cells might take up virions as the blebs retract.

### RSV may fuse its envelope with either the plasma membrane or the endosomal membrane, depending on the temperature of viral adsorption

**Figure 2:**
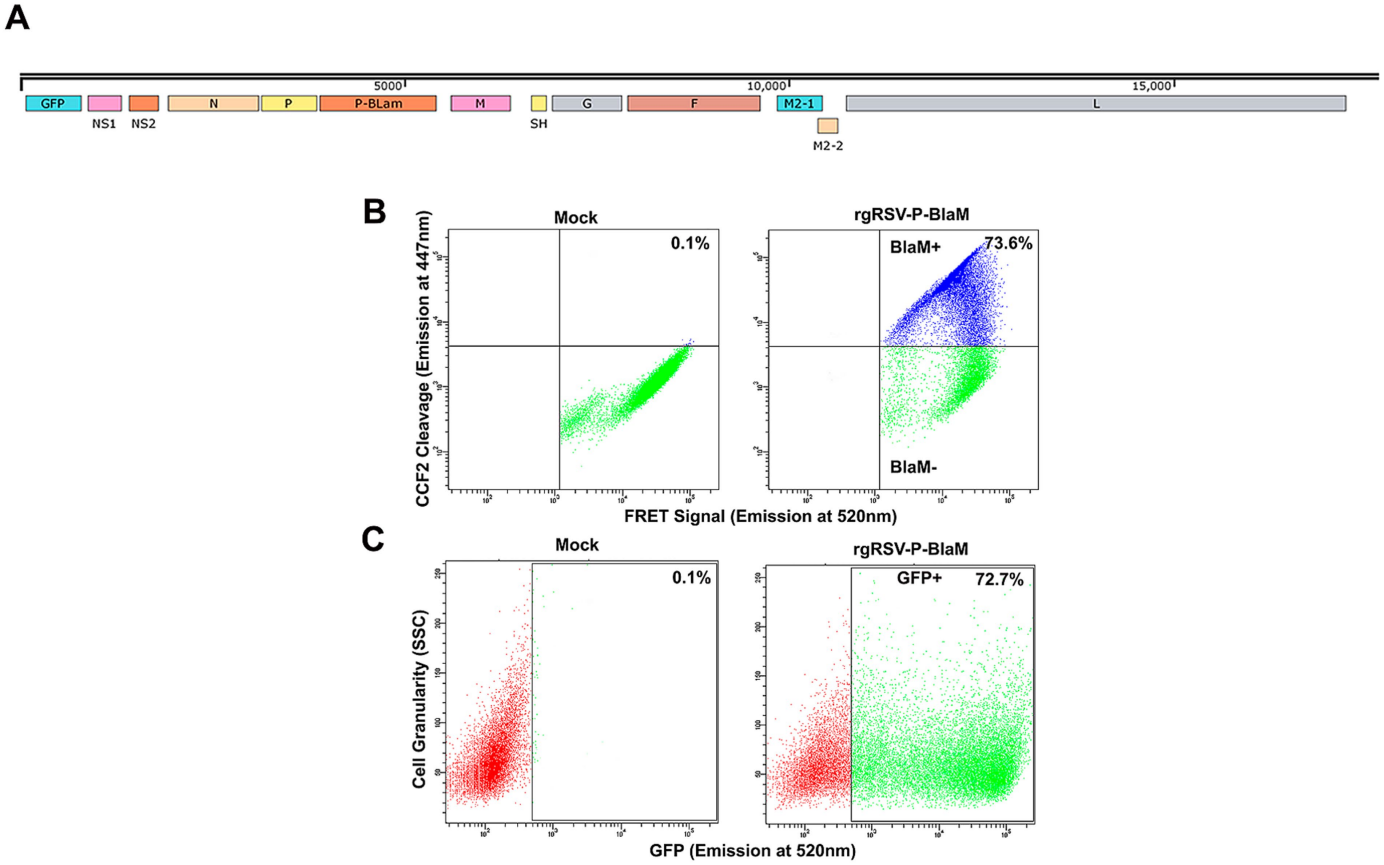
Characterization of rgRSV carrying chimeric protein P-β-lactamase. **(A)** Map of the rgRSV-P-BlaM genome. The gene encoding P-BlaM was inserted between the genes encoding the P and M proteins. **(B)** Evaluation of beta-lactamase activity in NHBE cells. After 2 h of rgRSV-P-BlaM inoculation, cells were uploaded with CCF2 for 3 h at 17°C. Then, FRET activity was evaluated by flow cytometry. The upper quadrants of the cytometry plots (mock vs. rgRSV-P-BlaM) show cells with BlaM activity disrupting the FRET signal. **(C)** Quantification of infected cells using GFP expression as a proxy for infection in rgRSV-P-BlaM-exposed NHBE cells. NHBE cells were exposed to the same viral inoculuous dose that were used to assess BlaM activity. GFP^+^ cells were detected 16 h after infection using flow cytometry.

We used an engineered RSV as described by De Ávila-Arias et al. (2024), which carries the enzyme beta-lactamase (BlaM) to assess viral entry. This method was previously used to explore HIV entry (Cavrois et al., 2002; Miyauchi et al., 2009). The RSV P gene was fused to BlaM, and the resulting P-BlaM gene was inserted downstream of the P gene in the RSV genome, which also contains the gene encoding GFP (rgRSV-P-BlaM) (Figure 2A).

Undifferentiated bronchial epithelial cells were inoculated with rgRSV-P-BlaM and virus entry was allowed to occur for 2 h at 37°C. The cells were then loaded with CCF2-AM, a fluorochrome with a beta-lactam ring linking a hydroxycoumarin and a fluorescein moiety. The BlaM protein delivered by the virion cleaves the beta-lactam ring, destroying the FRET signal and releasing the fluorescent hydroxycoumarin (Figure 2B), allowing us to identify those cells in which viral entry had occurred. To prevent further virus entry, CCF2 loading and concomitant cleavage by BlaM was performed at 17°C for 3 h (Figure 2B). We titrated rgRSV-P-BlaM, and measured BlaM activity at 3 h or GFP expression at 16 h. The percentage of cells with BlaM activity (73.6%) mirrored the percentage of cells expressing GFP (72.7%) (Figure 2C). Virus titer estimated by beta-lactamase activity, was 6.30 × 10^5^ FFU, while the titer estimated through GFP expression was 1.01 × 10^6^ FFU, as previously reported in De Ávila-Arias et al. (2024).

In general, two different approaches have been used to synchronize viral entry or infection: (1) spinoculation; and (2) attaching the virus to the cell membrane at temperatures not conducive to fusion.

Regarding spinoculation, Guo et al. (2011) cautioned against using spinoculation as a means to synchronize virus binding to cells. Spinoculation itself exerts biophysical and biochemical effects on cells that make them more susceptible to infection. Moreover, Guo et al. (2011) suggested that “some of the spin-induced cellular permissiveness may be beyond the natural capacity of an infecting virus.” In addition Jones et al. (2017), who demonstrated that Dynamin-2 stabilizes the HIV-1 fusion pore at the plasma membrane, avoided spinoculation because this technique induces changes in small GTPase activity that could disrupt actin cytoskeletal regulation with a potential knock-on effect on endocytosis.

Therefore, we followed the general approach of binding virions to the cell membrane at temperatures that either greatly slow or prevent the triggering of the fusion mechanism. Although the standard protocol binds virions to cells at 4°C, there are reports in the literature of synchronizing entry by binding virions at higher temperatures. Herold et al. (2014) synchronized binding of Vpr BlaM-HIV-1 at 22°C to assess HIV-1 entry into primary T cells. In addition, Fuller and Spear (1987) bound HSV-1 at 29°C.

A two-pronged approach made it possible to determine which cell membrane fused with the virus envelope. Virus attachment to the cell surface was carried out by incubation at 4°C or 22°C for 1 h. Then, unbound virions were washed away with HBSS. After warming to 37°C, virus entry was blocked at different times by the addition of palivizumab (AB) or by temperature (TB), placing the cultures at 4°C. Palivizumab neutralizes RSV infection by preventing the triggering of the conformational change from the prefusion to the post-fusion conformation of the RSV-F protein required for exposure of the fusion peptide (McLellan et al., 2011; Zhao et al., 2017). As a result, it prevents fusion with the plasma membrane. The TB protocol would block fusion of the virus envelope with any of the cell membranes (Miyauchi et al., 2009). Cold temperature reduces the lipid membrane fluidity.

If the viral escape rates of the TB protocol and the AB protocol were to overlap, this would indicate that the viral envelope was fusing with the plasma membrane. If the viral escape rate of the TB protocol were to be delayed relative to the AB protocol, this would indicate that the viral envelope is fusing with an endosomal membrane because it would take some time for the virus to reach the endosomal compartment where fusion occurs (Miyauchi et al., 2009).

**Figure 3:**
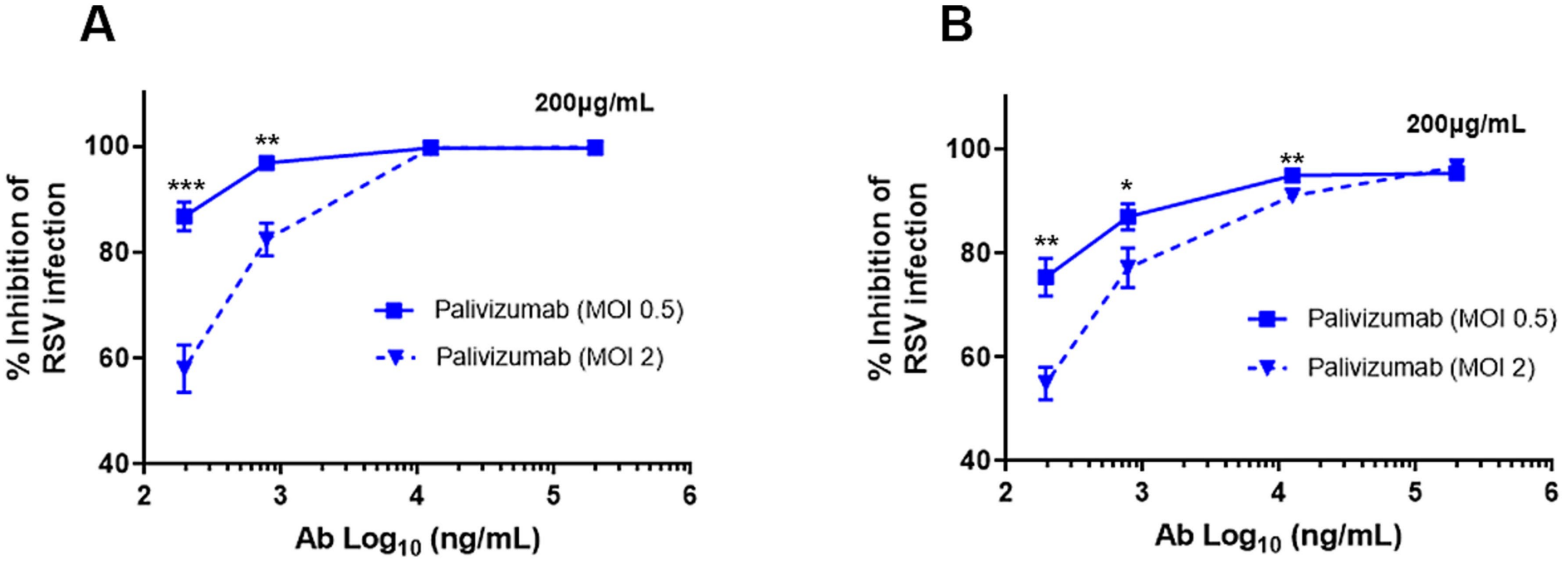
Palivizumab concentration required to neutralize RSV. **(A,B)** The following concentrations of palivizumab were tested: 200, 12.5, 0.78, 0.195, and 0.048 μg/mL and their Log_10_ transformation were plotted on the *x*-axis. Two infectious doses of RSV were tested (0.5 and 2.0 MOI). **(A)** RSV aliquots were incubated with the palivizumab at the aforementioned concentrations for 1 h at 37°C before adding to the NHBE cultures. **(B)** RSV aliquots were prebound to NHBE cells for 1 h at 22°C followed by the addition of palivizumab at the mentioned concentrations. Infection proceeded for 16 h and GFP expression was assessed by flow cytometry. Results are mean ± SEM of three independent experiments. An ANOVA followed by a one-way *t*-test was performed for statistical analysis. The level of significance was set at *p* < 0.05, ^*^p < 0.05, ^**^p < 0.01, and ^***^p < 0.001.

Palivizumab at the highest concentration tested (200 μg/mL) neutralized 99.7% of rgRSV-P-BlaM virions in suspension (Figure 3A) and 96.6% of cell surface-bound rgRSV-P-BlaM virions (Figure 3B). At this concentration, palivizumab was able to efficiently neutralize an infectious dose of 2 FFU/cell (Figure 3).

**Figure 4:**
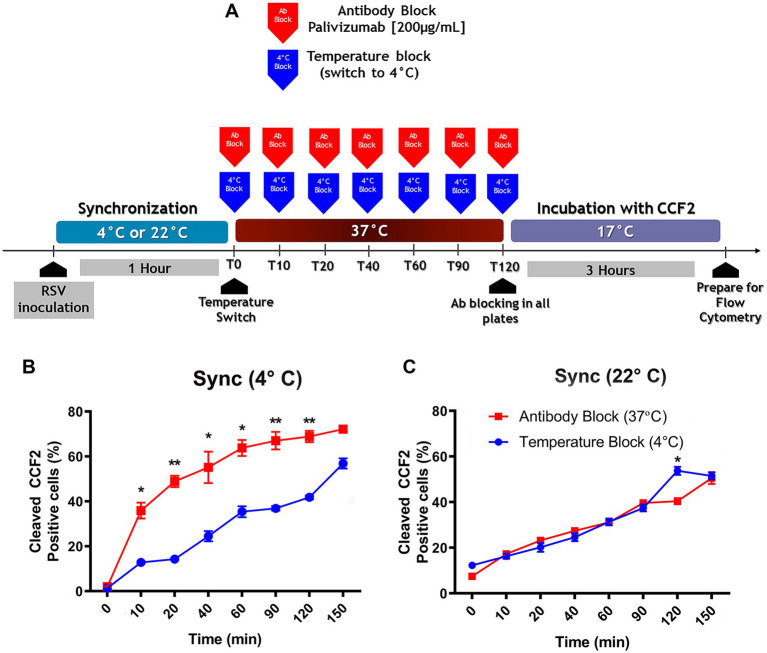
The preferred pathway used by RSV to enter NHBE cells depends on the temperature at which attachment took place. **(A)** An illustration of the experimental strategy that was followed to assess the influence of the viral attachment temperature on the preferred entry route of RSV into NHBE cells. **(B,C)** Kinetics of RSV entry after viral attachment at either 4°C **(B)** or 22°C **(C)** for 1 h on NHBE cell cultures. After switching to 37°C, we stopped virus entry at different times, by either antibody block (palivizumab, 200 μg/mL, blue squares) or temperature block (4°C, red circles). After washing, cells were loaded with CCF2-AM at 17°C for 3 h. The plots showed the percentage of cells positive for CCF2 cleavage due to BlaM at each time. Results are mean ± SEM of three independent experiments. An ANOVA followed by a one-way *t*-test was performed for statistical analysis. The level of significance was set at *p* < 0.05, ^*^p < 0.05, ^**^p < 0.01, and ^***^p < 0.001.

A schematic diagram of the protocol to compare the effects of is shown in Figure 4A. When RSV was attached to the cell surface at 4°C, the virus escape rate observed in the TB protocol was lower than that in the AB protocol (Figure 4B), indicating that attachment at 4°C leads to virus uptake by endocytosis. The fraction of internalized virions that were not fused was estimated by subtracting the percentage of cells showing BlaM activity in AB and TB at each time point. The percentage of internalized but unfused virions ranged from 25 to 30% at different times, as evidenced by TB.

When virions were bound at 22°C, the viral escape rates overlapped in both protocols (Figure 4C). This indicated that the cell surface-bound virions had fused their envelope to the plasma membrane. Therefore, the temperature chosen for synchronization influenced whether RSV was taken up by endocytosis or fused to the plasma membrane.

It is possible that RSV may have reached a temperature-arrested intermediate state (TAS) at 22°C, facilitating its viral escape from palivizumab upon warming to 37°C. Using an experimental design similar to the one we describe in Figures 4A–C, De La Vega et al. (2011) found that HIV pseudoviruses acquired resistance to the fusion inhibitor more rapidly than those preincubated at 4°C. However, we found that rgRSV-P-BlaM bound at 4°C escaped much faster than virions adsorbed at 22°C (Figure 4B vs. Figure 4C); consequently, this temperature did not induce TAS for RSV.

**Figure 5:**
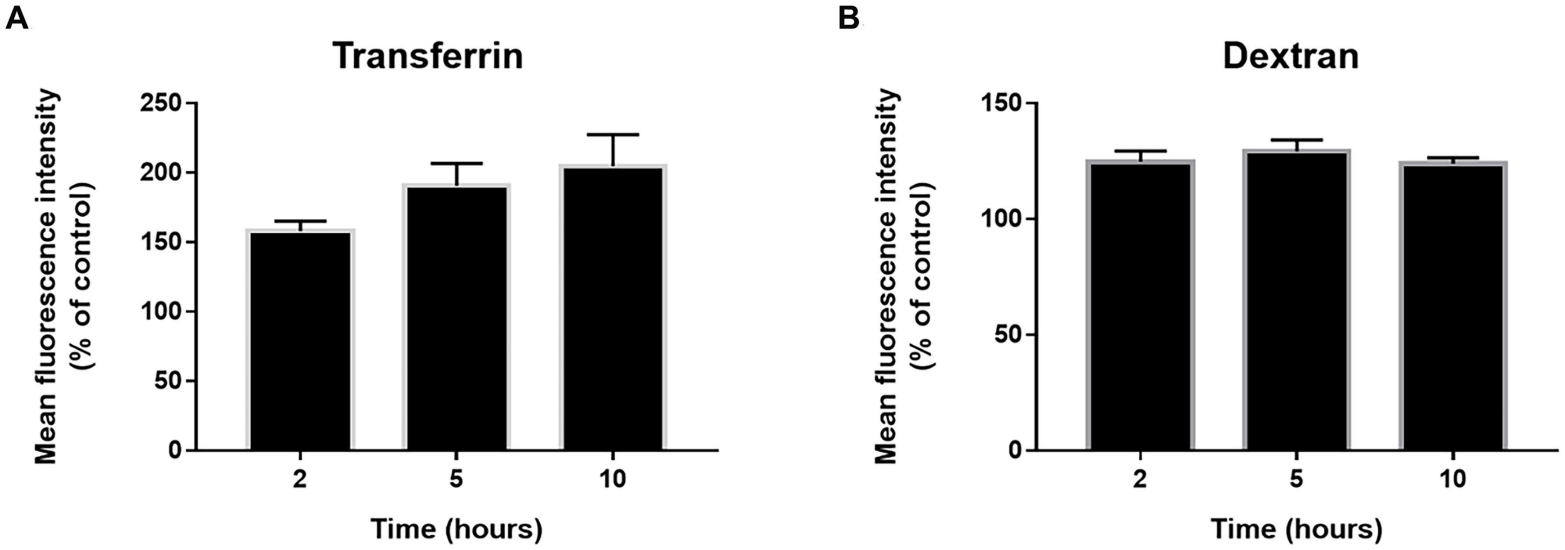
Endocytosis state at 22°C. The cells were incubated with phRodo red transferrin **(A)** and dextran **(B)** at 22°C for 2, 5, and 10 h. Then the cells were kept on ice and the fluorescence signal was determined by flow cytometry. The results are the mean ± SEM of three independent experiments and the mean fluorescence intensity was normalized to control cultures incubated with the tracers for 30 min at 37°C. An ANOVA test was performed for statistical analysis. The level of significance was set at *p* < 0.05, ^*^p < 0.05, ^**^p < 0.01, and ^***^p < 0.001.

Incubation at 22°C might prevent endocytosis in primary cultures of NHBE cells. Consequently, we evaluate general endocytosis processes at 22°C. pHrodo-labeled transferrin and dextran were used to evaluate clathrin-dependent endocytosis and pinocytosis/macropinocytosis, respectively. pHrodo is a fluorescent dye that shows little to no fluorescent signal at neutral pH but fluorescent brightly in an acidic environment such as late endosomes or lysosomes. This allows us to conduct the assays without preincubation at 4°C or the requirement of a negative control. Cell cultures were incubated with the respective tracers while kept at 22°C. The baseline was established by incubating cultures with the tracers at 37°C for 30 min. The 37°C incubation should not be considered a positive control, only a reference point. Both clathrin-dependent endocytosis (Figure 5A) and pinocytosis/macropinocytosis (Figure 5B) remained active at 22°C.

Since there are other endocytic pathways in addition to clathrin-dependent endocytosis or macropinocytosis, we addressed the question of whether cells could internalize virions at 22°C.

**Figure 6:**
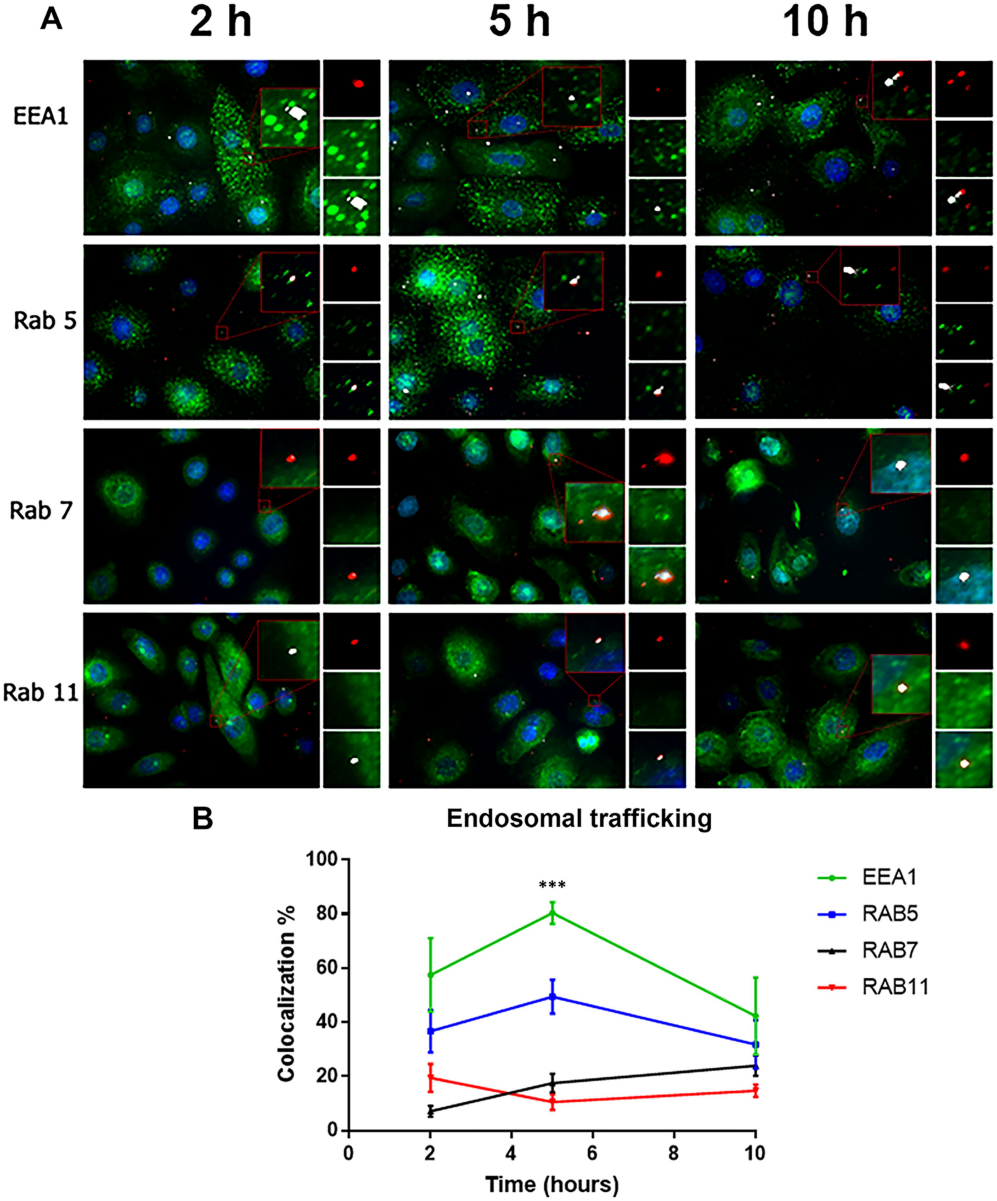
Colocalization of virions with the different endosomal compartments. **(A)** Cells were incubated with RSV (2 infectious virus particles per cell) at 22°C at different times (2, 5 and 10 h). Subsequently, the cells were fixed with PFA (4%) and permeabilized with saponin (0.05%). The RSV was detected using a primary antibody against the N protein. The signal of virions was enhanced by (TSA) and was detected in the rhodamine channel. The endosomal compartments were identified using rabbit primary antibodies against the respective markers, followed by incubation with Alexa 488-conjugated goat anti-rabbit secondary antibodies. Images were taken with an Axio Observer Z1. **(B)** Summary of the distribution of virions with the different endosomal compartments by time. The graph shows the percentage of colocalization with the different endosomal markers in the different times. The results are the mean ± SEM of three independent experiments. An ANOVA followed by a one-way *t*-test was performed for statistical analysis. The level of significance was set at *p* < 0.05, ^*^p < 0.05, ^**^p < 0.01, and ^***^p < 0.001.

RSV endocytosis and endosomal trafficking in cultured cells was assessed after virus adsorption at 22°C. Virions were probed by a mouse monoclonal anti-RSV antibody whose specific binding was detected by Tyramide Signal Amplification (TSA). The endosomes were identified using mouse monoclonal antibodies that recognize early endosomes (EEA1), primary endosomes (Rab5), recycling endosomes (Rab11), and late endosomes (Rab7). RSV virions (red) were detected inside all endosomal compartments (green) (Figure 6A). In Supplementary Figure S2, the corresponding negative controls for the TSA are shown to demonstrate the absence of any false labeling.

According to Heider and Metzner (2014), the ratio of total to infectious viral particles can reach 1,000 total particles per 1 infectious viral particle for RSV. Consequently, the total number of viral particles can range from 10 to 1,000 per infectious particle, and for the purposes of this assay, each cell of the monolayer may have been exposed to between 20 and 2,000 viral particles during the incubation period. Trypsin/EDTA was used to remove bound but unfused virions prior to labeling. A low number of RSV-N-associated signals per cell supports the success of the trypsin/EDTA treatment in removing unfused virions from the cell surface (Figure 6A).

We imaged 320 cells per experimental condition inquiring the colocalization of RSV with the endosomal marker. At 2, 5, and 10 h, the number of RSV-N-associated signals per cell was 1.61 ± 0.47, 1.61 ± 0.36, and 1.68 ± 0.37, respectively. After 2 h incubation at 22°C, RSV-N signal colocalized with the respective endosomal compartment as follows: 57.45 ± 13.61% of the RSV signal colocalized with early endosomes; 36.7 ± 7.84% of the RSV signal colocalized with primary endosomes; 19.44 ± 5.21% of the RSV signal colocalized with recycling endosomes, and 7.09 ± 2.06% of the RSV signal colocalized with late endosomes (Figure 6B and Supplementary Table S1). Overall, cells incubated at 22°C take up virions and maintain endosomal trafficking. We estimated the *p*-value of Costes included in Coloc-2 to determine whether the colocalization of RSV-N signals with the endosomal markers was fortuitous. If Costes’ *p*-value is >0.95, then, the probability of a spurious colocalization signal is less than 5%. A set of random images was selected to represent the three time points (2 h, 5 h, and 10 h) for each endosomal marker. We established the RSV-N signals as regions of interest (ROI). The RSV-N associated red signal was randomized using a point spread function (PSF) of 1, following Costes’ significance test. We found that the Costes’ *p*-value was 0.98 ± 0.02. In addition of the statistical analysis, the following reasons give additional support:

1. Although the endosomal markers always outnumbered the RSV-N-associated signals, the RSV-N-associated signals were distributed according to the expected trend of endosomal traffic maturation.
2. The average % of RSV-N associated signals distributed per endosomal compartment showed variations at each of the incubation times. In addition, RSV-N-associated signals increased their distribution in Rab7-positive endosomes at 10 h, which correlates with the expected maturation of endosomal trafficking. At longer incubation times, RSV-N associated signals are expected to accumulate in late endosomes.
3. There is no need to anticipate an increase in the number of distinct endosomal compartments showing RSV-N associated signals over time. Krzyzaniak et al. (2013) reported that the total number of RSV-containing spots decreased over time, which may be explained by the accumulation of multiple viral particles in a vacuole that appears as a single spot under confocal microscopy. This phenomenon is a consequence of homotypic fusion and has been reported to occur in each of the different EEA1^+^, Rab5^+^, Rab7^+^ or Rab11^+^ endosomal compartments (Mills et al., 2001; Zerial and McBride, 2001; Koike et al., 2024; Savina et al., 2005).
4. Endosomes can have two different endosomal markers during their maturation process. Rab5 can be found on early and primary endosomes. EEA1 can colocalize with Rab5 on early endosomes but also on Rab7-negative late endosomes. Rab7 primarily marks late endosomes and lysosomes (van der Beek et al., 2022).

A functional endocytosis assay was performed according to the guidelines of Herold et al. (2014) (Supplementary Figure S3). Palivizumab (200 μg/mL) was used to neutralize the virions that had not reached palivizumab-inaccessible compartments after 2, 5, and 10 h of incubation at 22°C. After the respective incubation time at 22°C, the cell cultures were warmed up to 37°C and the infection was allowed to progress for 18 h. The expression of GFP was used as a reporter for the infection. Palivizumab neutralization was expected to be as effective as trypsin treatment. Although there was a consistent low number of RSV-N signals inside cells, the fraction of GFP-expressing cells increased over time in each of the time points tested. This suggests that virions that were able to reach palivizumab-inaccessible compartments fused their envelopes after warming the cultures to 37°C. Thus, endocytosis was functional at 22°C.

### Temperature pulse, with neutralizing antibody chase

As shown in Figure 4C, BlaM signal was detected in 7 to 10% of the cells that took up CCF-2 at the time of warming to 37°C. Either these virions fused with the plasma membrane even at the suboptimal temperature of 22°C, or endocytosed virions may have fused to deliver BlaM to the cytosol.

**Figure 7:**
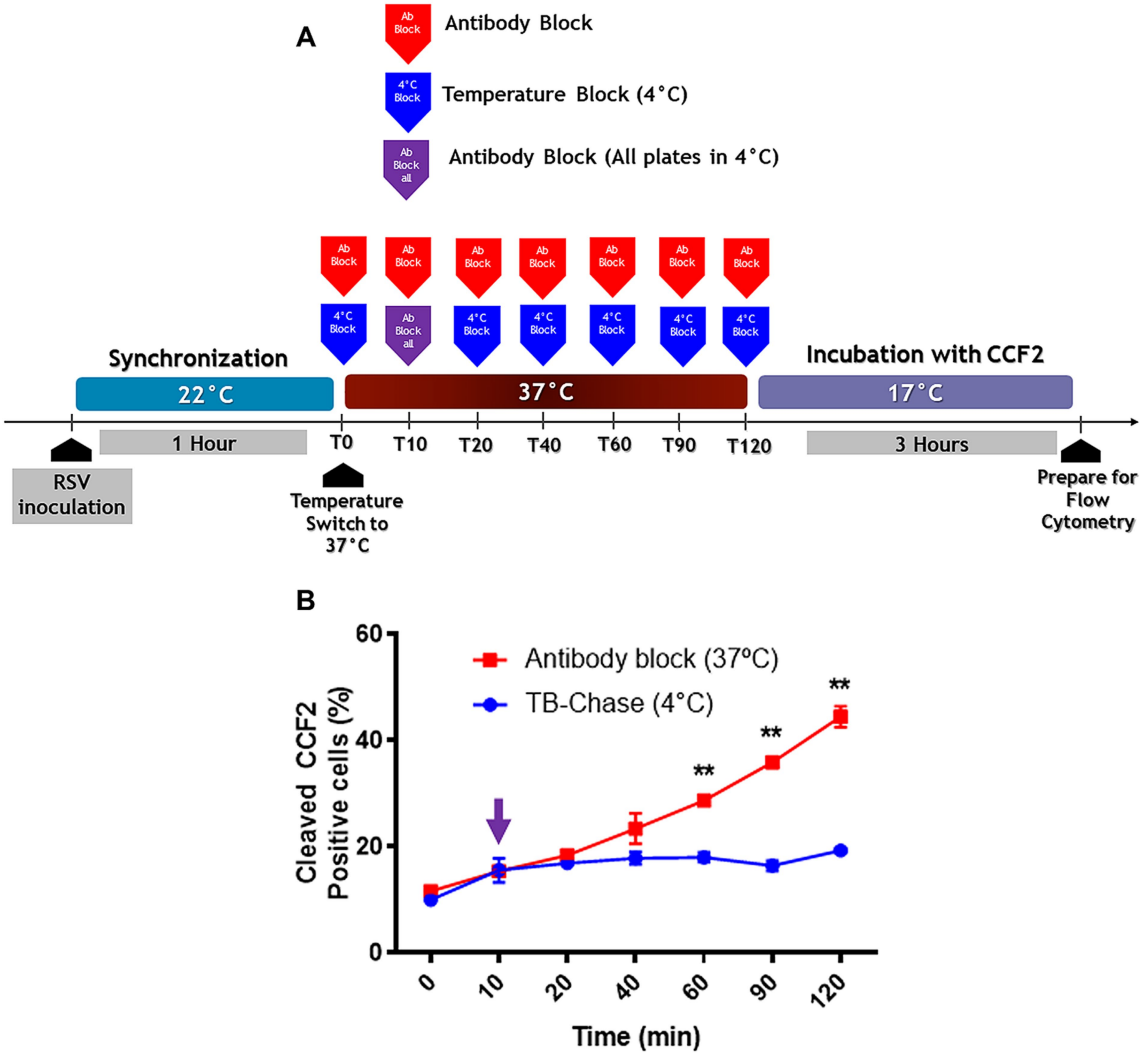
The TB-chase assay shows virions that attach to cells at 22°C enter the cell cytoplasm through the plasma membrane. **(A)** An illustration of the experimental strategy that was followed for TB-chase assay to determine whether virions inside endosomes contribute to infection. In **(B)**, the TB-chase assay showed a flat line (blue circles) indicating that RSV virions within endosomes contributed no infectivity after binding at 22°C. Results are the mean ± SEM of three independent experiments. An ANOVA followed by a one-way *t*-test was performed for statistical analysis. The level of significance was set at *p* < 0.05, ^*^p < 0.05, ^**^p < 0.01, and ^***^p < 0.001.

Given that there is basal endocytosis at 22°C and that we found virions inside the endosomes, how much do these virions contribute to the percentage of cells showing a positive signal due to BlaM? Hence the pulse-chase design; the virions were adsorbed to the plasma membrane for 1 h at 22°C. At the end of adsorption, all cultures were placed at 37°C. After 10 min, the set of cultures was divided into 2 arms, one following the AB block protocol (Figure 7, red line) and the other following the pulse-chase design (Figure 7, blue line). In the pulse-chase arm, all cultures received palivizumab 10 min after synchronization. A set of 3 cultures from the pulse-chase branch were immediately refrigerated to 4°C. This procedure was then repeated every 10 min for sets of 3 cultures from the pulse-chase arm. Once 2 h has lapsed, all cultures from both protocols were loaded with CCF2-AM at 17°C (Miyauchi et al., 2009).

If endocytosis is the preferred pathway for RSV to deliver its contents to the cytosol, an increase in BlaM signal would be expected at any time after neutralization of cell surface-adsorbed virions with palivizumab during the chase assay. However, there was no increase in BlaM signal even 120 min after warming to 37°C (Figure 7B).

### Screening for the identification of drugs that target early steps of RSV infection

We evaluated several drugs known to interfere with endocytosis for their effects on RSV infection using dose-response inhibition assays. The following drugs targeting different endocytic pathways were used in dose-response assays: Dynasore (dynamin inhibitor), latrunculin A (actin depolymerization), cytochalasin D (actin depolymerization), jasplakinolide (stabilizes preformed actin filaments *in vitro* and inhibits their disassembly), Y27632 (RhoA inhibitor), NSC23766 (Rac1 inhibitor), Casin (Cdc42 inhibitor), Wiskostatin (N-Wasp inhibitor), LY294002 (PI3K inhibitor), Wortmannin (PI3K inhibitor), Edelfosine (PI-PLC inhibitor), U73122 (PI-PLC inhibitor), EIPA (Na^+^/H^+^ exchanger inhibitor), and Ciliobrevin A (dynein inhibitor).

**Figure 8:**
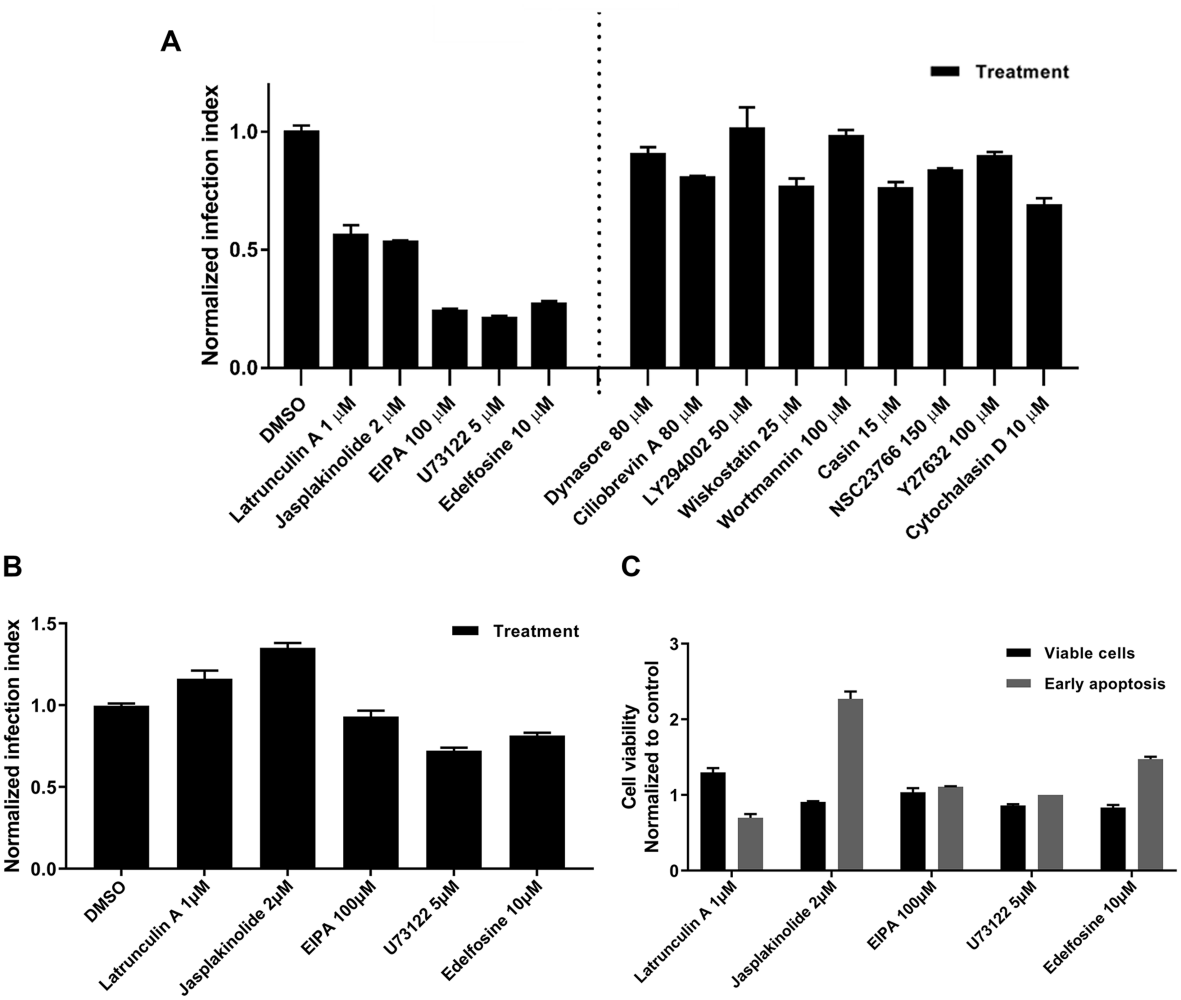
Screening of inhibitors targeting the early steps of the virus infection. **(A)** NHBE cells were exposed to a range of concentrations per each tested drug for 1 h before inoculating the respective cultures with rgRSV at a viral dose of 2 FFU per 10 cells. The virus infection at 37°C proceeded for 2 h in the presence of the respective inhibitor. After removing unabsorbed virions by washing with HEPES-based saline solution (HBSS), unfused virions were inactivated with cold trypsin (0.0025 mg%) for 8 min at 4°C. After washing trypsin away, infection was allowed to proceed for 16 h at 37°C before determining the percentage of GFP^+^ cells by flow cytometry. **(B)** Assessment of post-entry inhibitory action of each positive hit. NHBE cells were infected with rgRSV at 2 FFU per 10 cells for 2 h at 37°C. Unadsorbed virions were washed away using HBSS and unfused virions were inactivated with cold trypsin. The most effective concentration at inhibiting viral entry was tested per each positive hit for 3 h at 37°C. Then, the inhibitors were washed away using HBSS and fresh BEGM media were provided to cell cultures. Infection was allowed to proceed for 16 h and percentage of GFP^+^ cells was determined by flow cytometry. **(C)** Cytotoxicity evaluation of each positive hit. NHBE cells grown at 80% confluency were exposed to the most effective inhibitory concentration of each positive hit for 3 h at 37°C. Then, inhibitors were washed away using HBSS and respective cell cultures were incubated for 30 min in fresh BEGM media. Single cell solution was prepared from each of the conditions and incubated for 5 min at RT with Alexa-647-labeled annexin V and SYTOX blue. Cells were immediately analyzed using flow cytometry and the cell distribution in terms of being viable (double negatives), in early apoptosis (only positive for annexin V), or dead cells (positive for SYTOX blue) were determined.

Each drug was added to cell cultures 1 h before RSV inoculation and maintained during the first 2 h of infection. Infection was allowed to continue for 16 h, and RSV-infected cells were identified by GFP expression and counted. We have summarized the data by the lowest and highest concentrations of treatments that had inhibitory and no inhibitory effects on infection, respectively (Figure 8A). We used a cut-off of 50% reduction in RSV infection to identify hits in our drug screening. Drugs that inhibited infection are grouped to the left of the dotted line. Latrunculin A, jasplakinolide, EIPA, U73122, and edelfosin reduced the infection by at least 50% of that observed in DMSO-exposed cell cultures (Figure 8A). Treatments that failed to inhibit infection are grouped to the right of the dotted line.

To rule out long-term drug-associated effects that interfere with virus replication, each drug was administered after the first 2 h of RSV infection and left in the medium for 3 h. The drug was then removed, and fresh medium was added to the cell cultures. None of the drugs reduced RSV infection by 50% of that observed in DMSO-treated cell cultures (Figure 8B) indicating they do not affect viral replication. In addition, there was no cell cytotoxicity due to late apoptosis or necrosis (Figure 8C).

### Time of addition assays suggest alternative mechanisms to endocytosis

**Figure 9:**
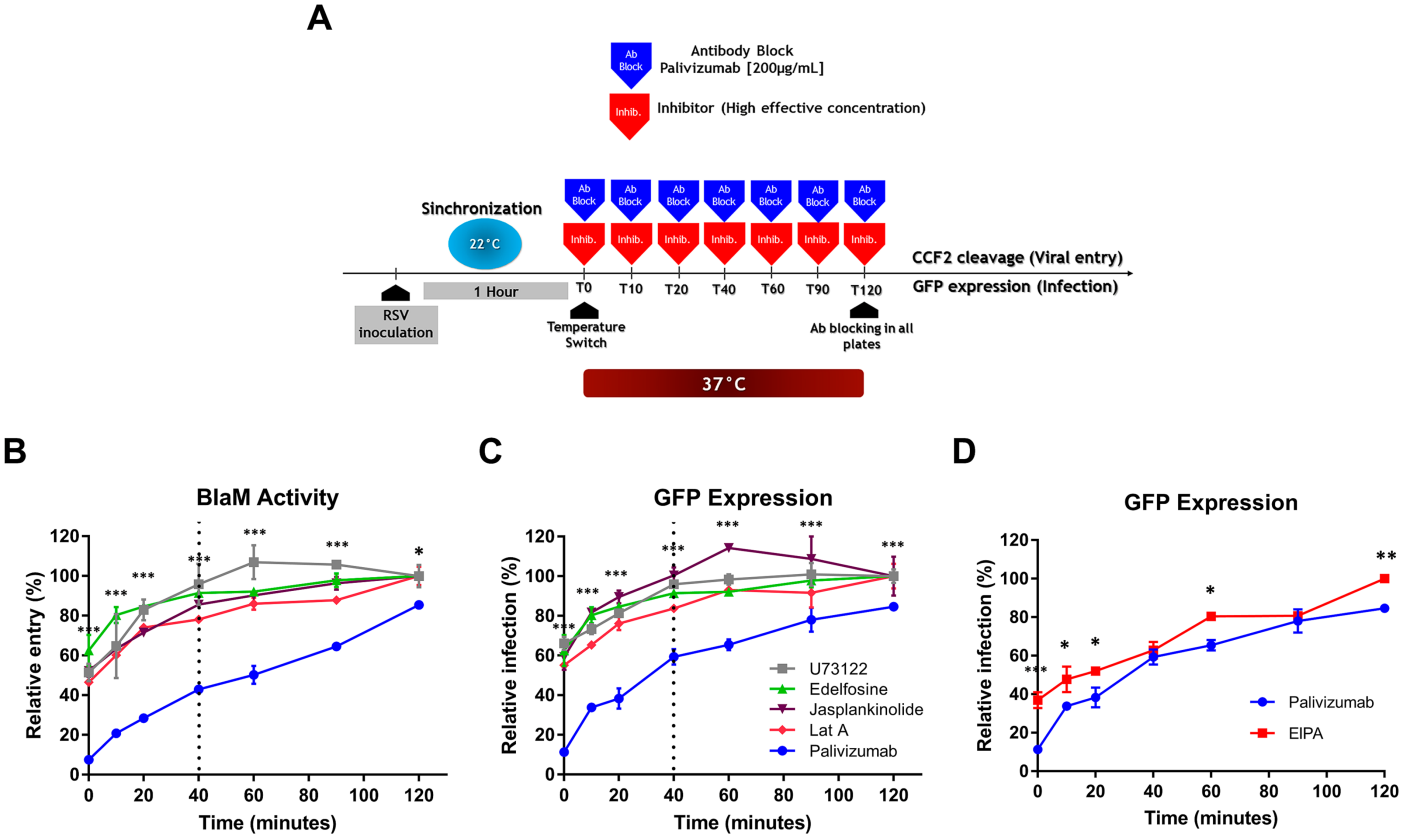
Time-of-addition assays suggest alternative mechanisms to endocytosis after synchronization at 22°C. **(A)** An illustration of the experimental strategy for the time-of-addition assay. CCF2 cleavage by BlaM evaluated viral entry. GFP expression assessed viral transcription and it is a proxy for infection. Flow cytometry was used to detect the respective fluorescent signal. In **(B–D)**, the time-of-addition assays showed that palivizumab was more efficient in preventing virus entry at each of the time points compared to inhibitors. **(B)** The time-of-addition assay for virus entry. **(C,D)** The time-of-addition assay for infection. In **(D)**, we set EIPA apart since its time-of-addition profile paralleled the entry kinetics using Ab blockade. Results are the mean ± SEM of three independent experiments. An ANOVA followed by a one-way *t*-test was performed for statistical analysis. The level of significance was set at *p* < 0.05, ^*^p < 0.05, ^**^p < 0.01, and ^***^p < 0.001.

Although these drugs are known to interfere with endocytosis, this does not necessarily mean that they prevent RSV infection in this way. Therefore, we performed ToA assays to investigate whether these drugs reduce RSV infection by targeting endocytosis. If the plasma membrane is where RSV fuses its membrane, ToA assays would show the same escape kinetics as the AB protocol because palivizumab interferes with viral fusion. On the other hand, if a drug’s ToA shows delayed escape kinetics compared to the AB protocol, it would imply that the drug is preventing the endosomal uptake required for RSV to reach the site where fusion would occur. A schematic representation of the procedure is shown (Figure 9A), which was conducted following the Miyauchi et al. (2009) approach.

We assessed viral escape by BlaM activity or GFP expression. EIPA showed very high fluorescence at the same wavelength as hydroxycoumarin. Therefore, we could not assess BlaM activity when cells were exposed to EIPA. None of the ToA of the drugs showed the same escape kinetics as was seen with the ToA of palivizumab (Figures 9B–D). All the drugs, except EIPA, rapidly lost their ability to prevent RSV escape; at least 50% of the cells showed BlaM activity or GFP expression when the cultures were shifted to 37°C, and they were no longer able to neutralize viral escape 40 min later (Figures 9B–D). In the case of EIPA, 35% of cells showed GFP expression at the time of warming to 37°C. However, the loss of sensitivity to EIPA followed a linear trend (Figure 9D). Palivizumab administered 40 min after temperature shift to 37°C failed to neutralize 50% of infection events as estimated by GFP expression (Figure 9C).

Although some of the drugs would not reach complete inhibitory effect shortly after administration, ToA approach would still be useful since it would serve as an introductory way to determine if the kinetics of RSV escape from these drugs would be similar to that determined by the AB protocol. There was no difference in the kinetic rates suggesting that all of them may be impairing fusion at the cell membrane (Supplementary Table S2; *p* = 0.305).

### Drugs that prevented RSV entry perturbed cell surface morphology

**Figure 10:**
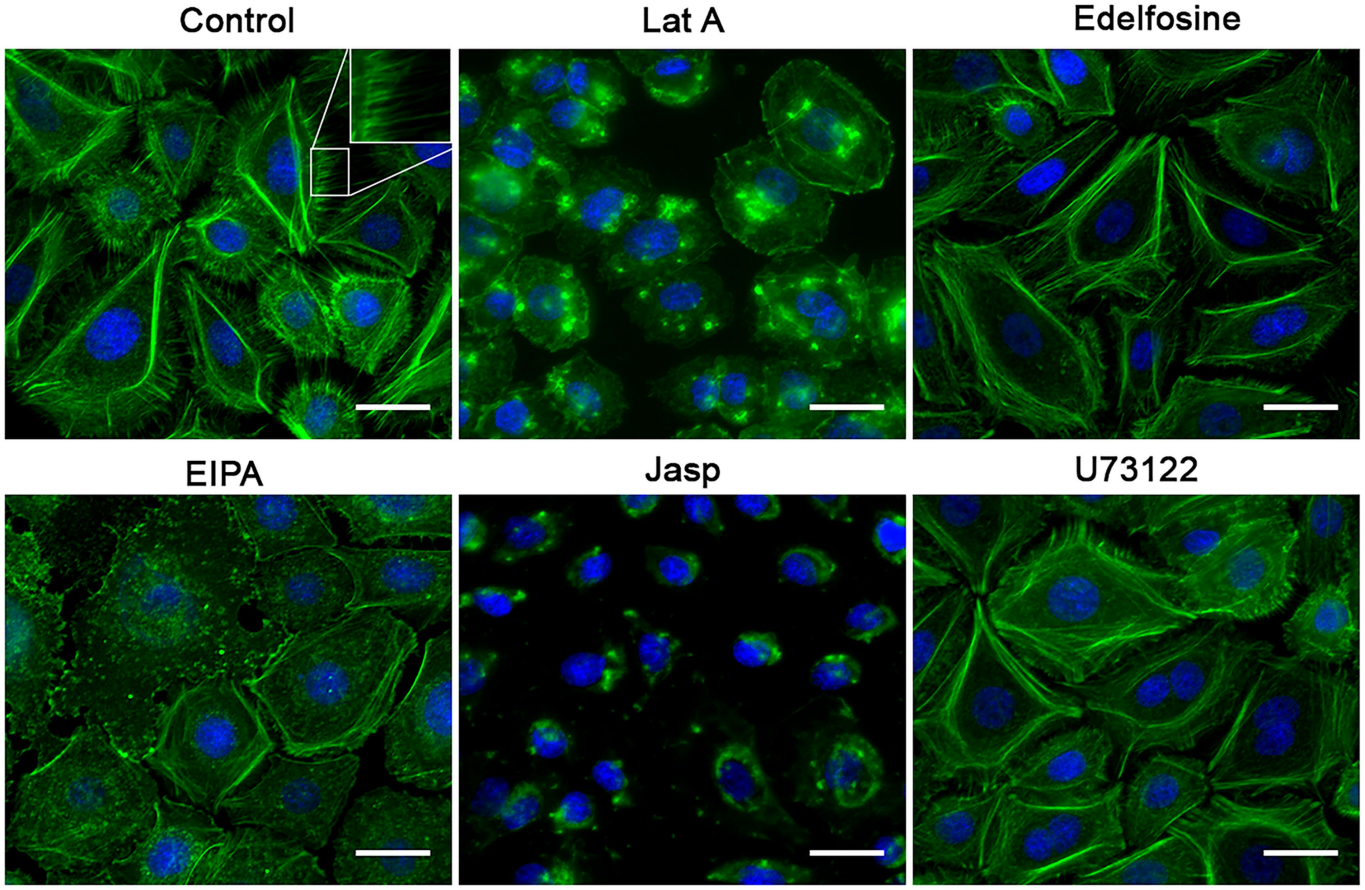
Effect of the inhibitors on the F-actin distribution. NHBE cells grown at 80% confluency were exposed for 3 h at 37°C to the most effective concentration of the positive hit previously identified. After washing with HBSS, the cell monolayer was fixed with 4% PFA at 4°C followed by cell permeabilization with 1% Triton X-100. Alexa-488-labeled phalloidin and DAPI were used to label F-actin and nuclei, respectively. A motorized, inverted fluorescence microscope (Axio-observer Z1) was used to visualize cells at 63× and images were taken using the AxioCam HR CCD. All positive hits disrupted F-actin distribution. All treatments significantly reduced the presence of filopodia. Cells treated with latrunculin A (Lat A) and jasplakinolide (Jasp) precipitated F-actin in clusters and showed an absence of stress fibers. Treatment with edelfosine and U73122 caused cells to increase the amount of stress fibers. The most distinctive feature in the EIPA-treated cells was the increase in F-actin signal at the cell boundary as if making the 3 cell border more visible.

**Figure 11:**
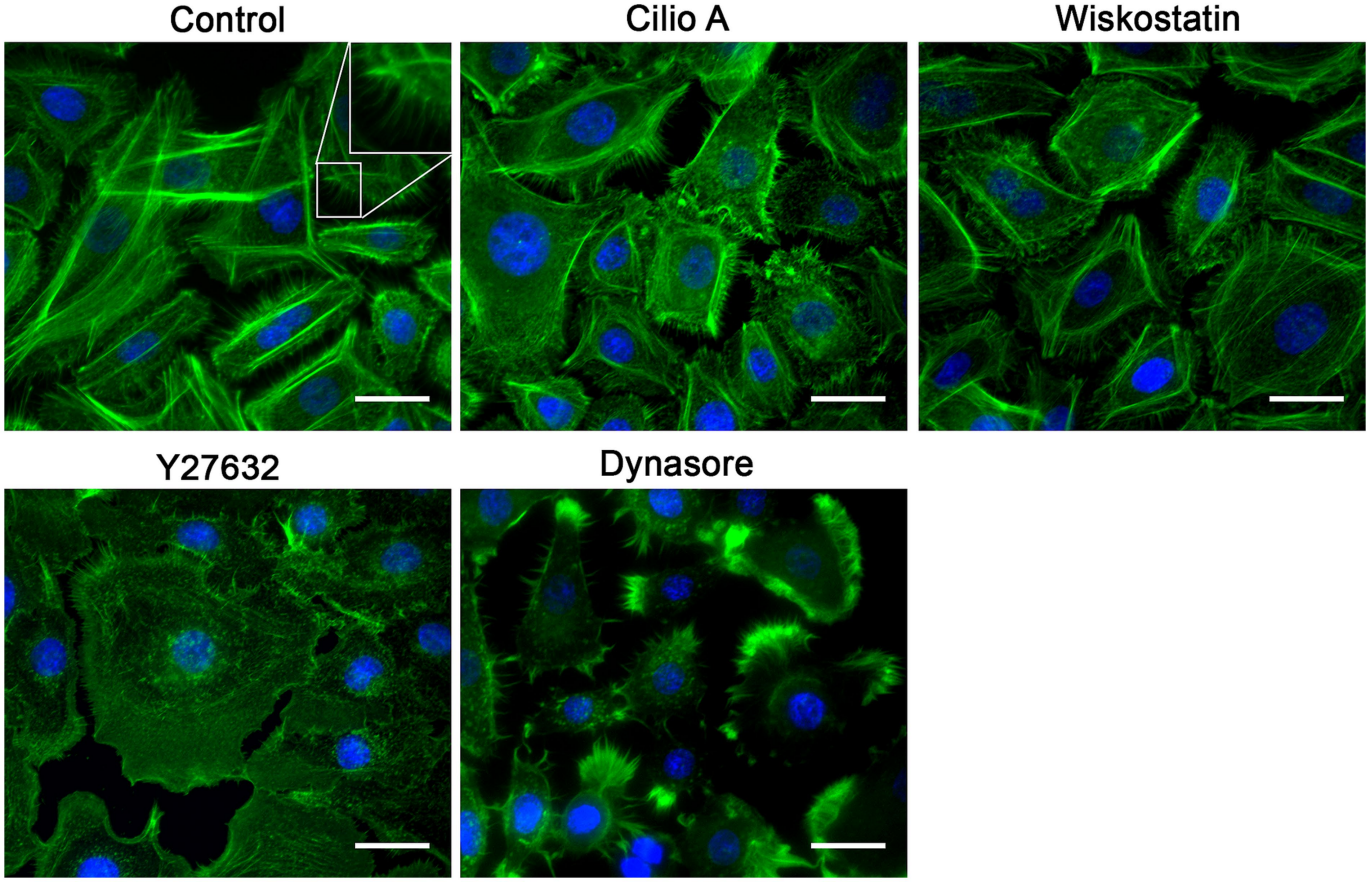
Effect of the molecules that did not affect the RSV infection on the F-actin distribution. NHBE cells grown at 80% confluency were exposed for 3 h at 37°C to the most effective concentration of the positive hit previously identified. After washing with HBSS, the cell monolayer was fixed with 4% PFA at 4°C followed by cell permeabilization with 1% Triton X-100. Alexa-488-labeled phalloidin and DAPI were used to label F-actin and nuclei, respectively. A motorized, inverted fluorescence microscope (Axio-observer Z1) was used to visualize cells at 63× and images were taken using the AxioCam HR CCD. All positive hits disrupted F-actin distribution. All treatments significantly reduced the presence of filopodia. Cells treated with latrunculin A (Lat A) and jasplakinolide (Jasp) precipitated F-actin in clusters and showed an absence of stress fibers. Treatment with edelfosine and U73122 caused cells to increase the amount of stress fibers. The most distinctive feature in the EIPA-treated cells was the increase in F-actin signal at the cell boundary as if making the cell border more visible.

Since these drugs were expected to reduce the RSV entry kinetics, we assessed the impact of these drugs on the actin cytoskeleton as a proxy for detecting changes on the actin-dependent plasma membrane morphology. Actin dynamics underlies plasma membrane curvature and tension. We used Alexa-488-tagged phalloidin to detect F-actin. All drugs that impair RSV infection were found to reduce cell membrane deformations, such as filopodia and lamellipodia (Figure 10) which correlates with a reduction in actin availability for the formation of these structures and would be indirectly associated with changes in membrane curvature. Particularly, an increase in the stress fibers was observed in cell cultures exposed to U73122 and edelfosine. As expected, F-actin collapsed when cell cultures were treated with latrunculin or jasplakinolide. Cultures treated with EIPA showed a marked increase in the F-actin distributed along the edges of the cell, suggesting an increase in the cortical actin. When we probed F-actin in cell cultures exposed to drugs that did not block RSV infection, membrane deformations—filopodia and lamellipodia—were preserved. Actually, dynasore-treated cell cultures showed a marked increase in filopodia (Figure 11).

## Discussion

Overall, the results of this study indicate that RSV can enter and infect undifferentiated NHBE cells via fusion with the plasma membrane. We found that warming to 37°C following viral attachment at 4°C caused a striking cell membrane response in the form of massive blebs. By some unknown mechanism, perhaps related to the plasma membrane blebs that form at 4°C, plasma membrane-adsorbed virions are taken into the cell by an unnatural endocytic mechanism, resulting in enhanced RSV infection. When the virions were adsorbed at 22°C, RSV fusion seemed to occur with the plasma membrane.

The fact that rgRSV-P-BlaM carries the preformed BlaM enzyme allowed us to perform the content-mixing experiments. Recombinant HIV-1 virions carrying BlaM-Vpr, designed by Cavrois et al. (2002), contributed to understanding how HIV-1 enters target cells and to identifying a set of molecules important for viral entry (Miyauchi et al., 2009; Herold et al., 2014; Putcharoen et al., 2012; Durham and Chen, 2016; Kondo et al., 2015; Cavrois et al., 2004). Pseudotyped lentiviruses expressing BlaM-Vpr allowed the identification of the mechanism of entry of Hepatitis C virus or Ebola virus. Virus-like particles carrying BlaM have also been useful for studying the entry of influenza A and henipavirus (Wolf et al., 2009; Lavillette et al., 2007; Lavillette et al., 2006; Yonezawa et al., 2005; Tscherne et al., 2010).

We found that synchronizing RSV infection at 4°C leads to erroneous conclusions about how RSV enter cells which may also be applicable to other viruses. In undifferentiated primary cultures of human bronchial epithelial cells, rapid warming to 37°C induces the formation of giant blebs protruding from the cell surface, which contain actomyosin rings. As the blebs retract, they might internalize plasma membrane-bound virions by macropinocytosis (Mercer and Helenius, 2009). In fact, Krzyzaniak et al. (2013) also demonstrated the formation of blebs upon warming to 37°C after RSV adsorption to HeLa cells at 4°C; and they also found that the macropinocytosis that follows bleb retraction is the mechanism by which RSV is taken up by HeLa cells. When the virus attached to the cell membrane at 22°C, no blebbing was observed upon warming to 37°C.

It is interesting that when virions were adsorbed to cells at 4°C, RSV fusion was significantly delayed relative to its escape from palivizumab, whereas there was no delay when virions were adsorbed at 22°C. The fact that preincubation of cells with RSV at 22°C resulted in similar virus escape rates in both TB and AB protocols implied that viruses fused their envelope to the plasma membrane. If the virions require intracellular furin to activate the prefusion form of the F protein as Krzyzaniak et al. (2013) reported, at least some delay would be seen, even more so if endocytosis becomes the rate-limiting step.

There is a potential concern that the TB and AB protocols cannot resolve kinetic differences of a few minutes where a virus rapidly fuses with an early endosome after internalization. San-Juan-Vergara et al. (2012) previously reported that the entire process of RSV covering endocytosis and fusion with the endosomal membrane takes 37 min in single virus tracking assays on NHBE cells. Although Krzyzaniak et al. (2013) did not conduct real-time tracking of single viral particles, they reported that double-labeled RSV virions adsorbed at 4°C fused with the membranes of Rab5-positive endosomes on average 50 min after internalization. Thus, the TB and AB protocols used in this study may have sufficient temporal resolution to distinguish between fusion with the plasma membrane and fusion with the endosomal membrane.

Krzyzaniak et al. (2013) found their viral preparations enriched in RSV F proteins cleaved only at FCS1, requiring an endosomal proteolytic cleavage at FCS2 to remove p27 still attached to the fusion peptide. Helenius’ team reported a morphological analysis of the virions they used for the Krzyzaniak et al. (2013) paper using electron cryotomography (Liljeroos et al., 2013). They found that most of their viral preparation consisted of viral particles with globular morphology and a small fraction of filamentous virions. In addition, they showed that all but the F proteins on the globular particles were in the post-fusion conformation. In the case of the filamentous virions, some were predominantly in the prefusion conformation, while others were almost exclusively in the postfusion conformation, as if there were a systematic conformational change from one form of F to the other. The fact that the virions in their viral preparation may have triggered the conformational change of most of the fully cleaved preF to the postF form may explain why these virions require a furin-like protease in the endosomal pathway to activate the prefusogenic form. Rezende et al. (2023) estimated that the F protein containing p27 was 22.5% of the F proteins exhibited on the envelope of sucrose-purified RSV/A/Bernett virions. Consequently, endosomal proteolytic processing of prefusogenic F proteins, as proposed by Krzyzaniak et al. (2013), would serve as an alternative entry mechanism for those virions that have triggered the conformational change of most of their trimeric, fully cleaved F proteins.

We would like to highlight the report by Alonas et al. (2014) in which they performed single particle tracking of filamentous RSV virions adsorbed on Vero cells. They labeled the genome of the virions using MTRIP probes. They found that the lipophilic carbocyanine DiOC did not efficiently label the filamentous virions. Only the spherical particles were labeled with DiOC. Interestingly, they concluded that RSV filamentous virions fused their envelopes with the plasma membrane based on the radius of gyration and velocity of each filament tracked. In addition, no endosomal marker was found where the filaments fused with the Vero cells.

Although we allowed the infection to continue for 3 days, we changed the medium on the second day and then collected only the virions released into the culture media during the last 20 h. We also scraped the RSV-infected cell monolayer. Finally, we used MgSO_4_ to stabilize RSV infectivity and stored the virions at temperatures below −135°C. Overall, it is possible that our viral preparation had a better representation of virions, either filaments or spheres, with trimeric, fully cleaved RSV F proteins.

Although we showed the presence of RSV inside the endosomes at 22°C, endocytosed virions seemed not to contribute to the BlaM signal in the pulse-chase experiment. However, it is possible that the number of endocytosed virions were not frequent enough for the BlaM signal. Reexamining the entry mechanism of multiple enveloped viruses synchronized after binding at 4°C would be of interest (Sánchez-Felipe et al., 2014; Tan et al., 2016; Cox et al., 2015; Gonçalves-Carneiro et al., 2017; Owczarek et al., 2018; Rossman et al., 2012; Nanbo et al., 2010; Rasmussen and Vilhardt, 2015; Hollidge et al., 2012; Carvalho et al., 2017; Hetzenecker et al., 2016).

Although edelfosine, U73122, latrunculin A, jasplakinolide, and EIPA reduced infection when administered before and during viral adsorption, these drugs affect the composition, curvature, or tension of the cell membrane, which may result in impaired fusion events at the plasma membrane (Ríos-Marco et al., 2011; Mollinedo and Gajate, 2021; Ausili et al., 2018; Melikyan et al., 1997; Burgdorf et al., 2010; Markosyan et al., 1999; Masereel, 2003). A negative curvature in the outer leaflet of the plasma membrane is required for the hemifusion stalk formation. Since edelfosine accumulates in the outer leaflet of the lipid bilayer (van Blitterswijk and Verheij, 2013), it will induce a positive curvature that interferes with stalk formation and subsequent hemifusion (Busto et al., 2008; Fanani and Ambroggio, 2023; Mahadeo and Prenner, 2020; Ailte et al., 2017). In contrast, a positive curvature in the inner leaflet of the lipid bilayer is required for fusion pore expansion (Smirnova and Müller, 2021).

The overall tension of the membrane and the tension at the boundary of the fusion pore contribute to the opening of the pore and to its expansion, respectively (Kozlov and Chernomordik, 2015). Decreasing the overall tension prevents the fusion pore from forming. Once the pore opens, its expansion depends on the presence of curvature-inducing proteins at the pore edge. PIP2 contributes to the dynamics of cortical actin and recruits proteins that induce membrane curvature (Nebl et al., 2000). These proteins reduce the local tension. Therefore, reducing the presence of such proteins at the edge of the fusion pore affects its ability to expand (Kozlov and Chernomordik, 2015). U73122 decreases the levels of phosphatidyl inositol 4,5-diphosphate (PIP2) in the inner leaflet (Burgdorf et al., 2010). In addition, the impairment of actin dynamics will interfere with the expansion of pore fusion (Kozlov and Chernomordik, 2015); therefore, the formation and/or expansion of fusion pores may be affected by latrunculin A and jasplakinolide (Markosyan et al., 1999). Since EIPA is an inhibitor of the Na^+^/H^+^ exchanger (Masereel, 2003), it prevents submembranous pH correction, resulting in impairment of actin dynamics and consequent interference with fusion pore expansion (Kozlov and Chernomordik, 2015). Interestingly, Ida et al. (2023) showed that EIPA smoothened the cell surface, which is consistent with our findings regarding the absence of cell membrane deformations such as filopodia and lamellipodia in cell cultures treated with EIPA.

For understanding how each inhibitor may be impairing RSV fusion with the plasma membrane, we would need a multidisciplinary approach of advanced light microscopy using FRET, FLIM-FRET and super-resolution microscopy. In addition, scanning ion conductance microscopic imaging on living cells will be required to determine how different inhibitors affect plasma membrane dynamics, as shown by Ida et al. (2023).

In conclusion, our data imply that RSV fuses its envelope with the plasma membrane. Virus adsorption to the cell membrane at 4°C generates artifacts due to the cellular response that follows the transition from 4°C to 37°C. For this reason, we suggest it is critical to use temperatures closer to 37°C, such as the one used in the present study. Even if a drug is known to block endocytosis, this is not sufficient to conclude that endocytosis is the preferred mechanism by which a virus infects cells. These drugs also interfere with the biophysical and biochemical properties of the cell membrane required for viral fusion. Future studies will further elucidate the entry mechanisms of RSV, including the evaluation of viral entry in well-differentiated airway epithelial cultures.

## Data Availability

The data presented in the study regarding the sequence of RSV-P-BlaM was deposited in the GenBank repository, accession number PV083665. In addition, the rest of the raw data supporting the conclusions of this article will be made available by the authors, without undue reservation.
