# Supplementary material for: Respiratory syncytial virus fuses with plasma membrane to infect primary cultures of bronchial epithelial cells
Source: Front Microbiol. 2025 Feb 26;16:1498955. doi: 10.3389/fmicb.2025.1498955 (PMC11911548; doi:10.3389/fmicb.2025.1498955)
Supplement: Supplementary file 1 [file Data_Sheet_1.pdf]

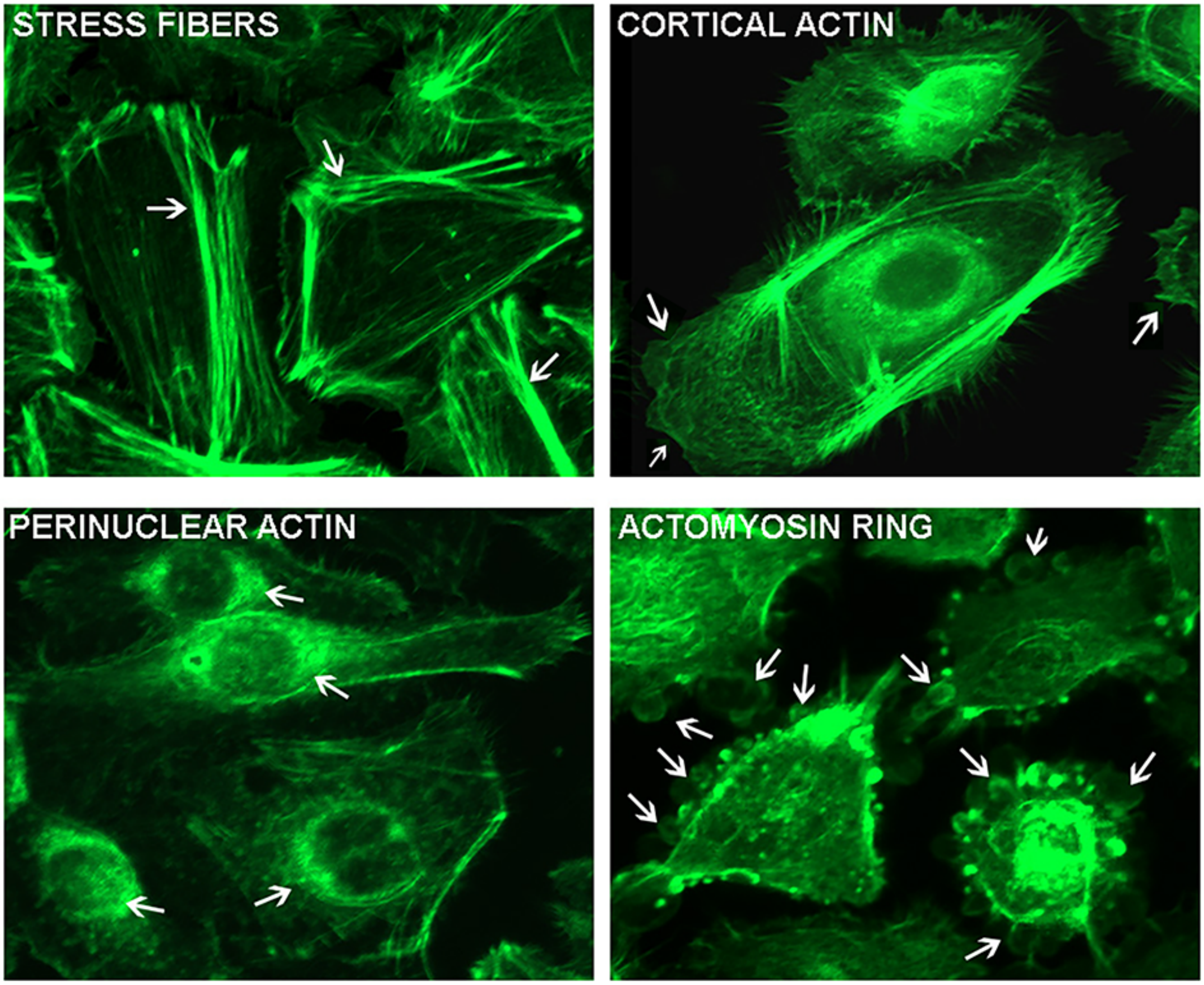

Supplementary Figure 1. F-actin-associated structures. Primary cultures of undifferentiated bronchial epithelial cells were grown in 8-well chamber coverslips to 80% confluency. Cells were fixed with 4% paraformaldehyde at 4°C for 20 min followed by permeabilization with 1% Triton at room temperature. Alexa-488-labeled phalloidin was used to label F-actin. A motorized, inverted fluorescence microscope (Axio-observer Z1) was used to visualize cells at 63x and images were taken using the Axiocam HR CCD.

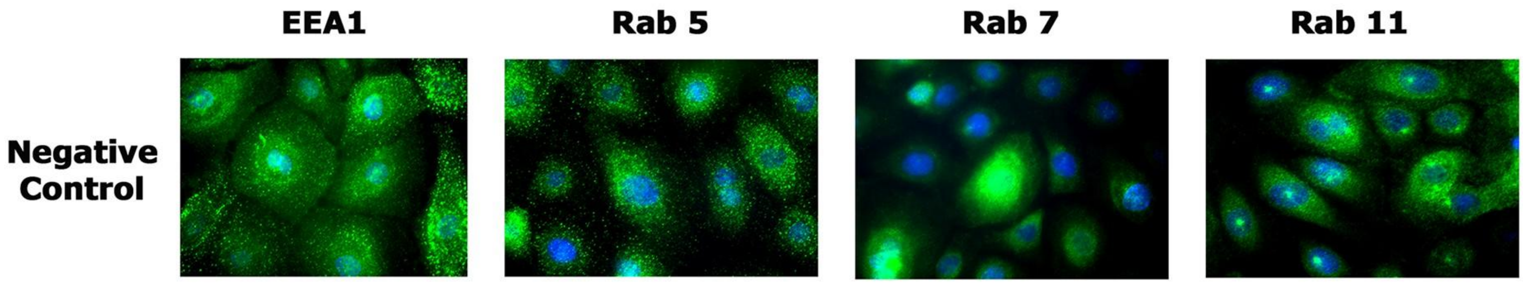

Supplementary Figure 2. This figure shows the negative control corresponding to Figure 6A. No TSA signal is detected in the absence of the primary mouse antibody against the RSV-N protein, confirming the absence of nonspecific signals.

Colocalization of RSV-N signals per Endosome						
	2 H		5 H		10 H	
**ENDOSOME**	% Average	SD	% Average	SD	% Average	SD
**EEA1**	57.45	13.61	79.2	5.7	42.5	11.3
**RAB5**	36.7	7.84	46.8	7.2	29.9	8.0
**RAB7**	7.09	2.06	19.5	4.9	24.5	4.4
**RAB11**	19.44	5,21	9.6	2.6	13.3	3.3

Supplementary table 1. The table shows the distribution of RSV-N associated signals per endosomal marker at the different times

Inhibitor	K
AB (Palivizumab)	0,030
U73122	0,061
Edelfosine	0,042
Jasplankinolide	0,040
Lat_A	0,055
EIPA	0,033

Supplementary table 2. The table shows the kinetic rate for each of the ToA assays corresponding to the drugs. The equation model for the rate constants was:

$$y(t) = \frac{L}{1 + e^{-k(t-t_0)}}$$

L is the maximum asymptote; **K** is the kinetic rate; **T_0** is the time in which the curve made the inflexion, or middle point of the curve

For the analysis, each inhibitor K was assessed in 5 levels. To determine if there are statistical differences, we used a one-way ANOVA; $\alpha = 0.05$.

H0: There is no statistical difference between the values of K corresponding to each inhibitor

H1: At least one of the inhibitors showed a different K value implying a different dynamic

Source	dof	Sum Sq	Mean Sq	F value	p-value
Inhibitor	4	0.002919	0.0007298	1.603	0.305
error	5	0.002276	0.0004552		

According to the p-value there is no difference in the kinetic rates suggesting that all of them may be impairing fusion at the cell membrane.

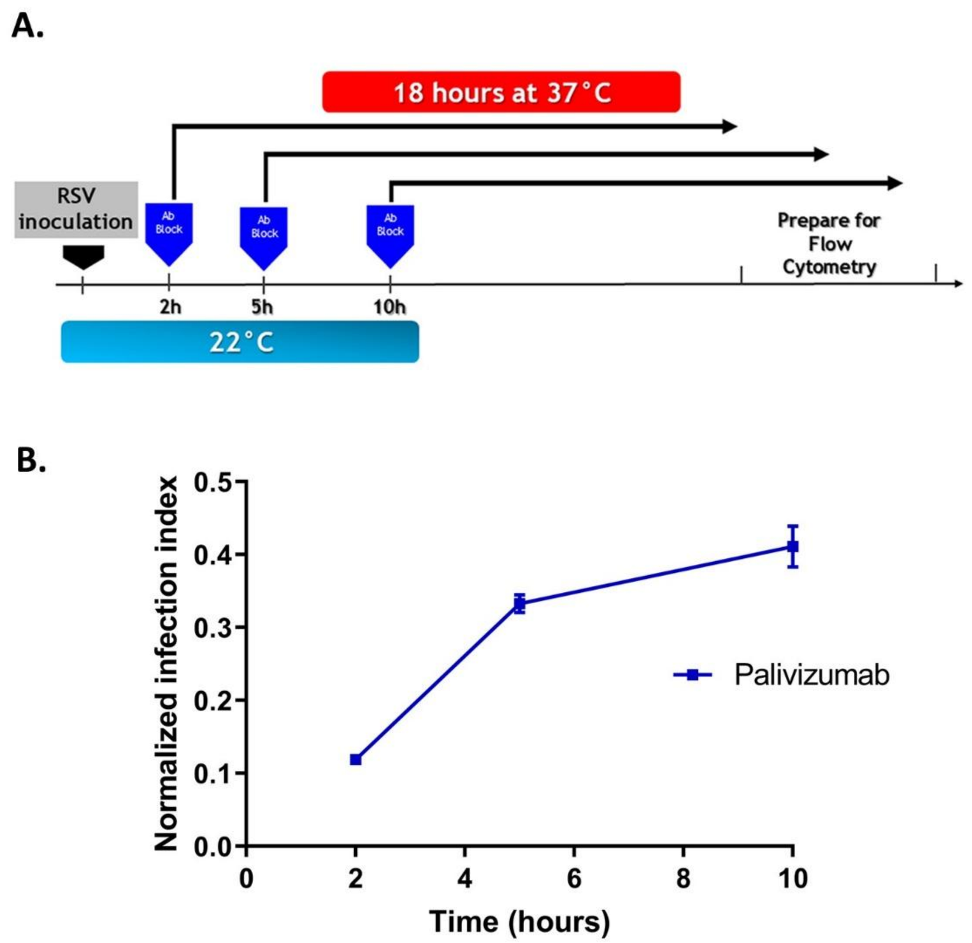

Supplementary figure 3. Endocytosis functional assay at 22° C. After incubation at 22°C for different time intervals (2, 5, and 10 h), virions that were still adsorbed to the cell membrane were neutralized with Palivizumab (200 µg/mL). Then, cell cultures were warmed up to 37 °C and the infection was allowed to progress for 18 h. The expression of GFP was used as a reporter for the infection.
